# Multivariate pattern classification of pediatric Tourette syndrome using functional connectivity MRI

**DOI:** 10.1111/desc.12407

**Published:** 2016-02-01

**Authors:** Deanna J. Greene, Jessica A. Church, Nico U.F. Dosenbach, Ashley N. Nielsen, Babatunde Adeyemo, Binyam Nardos, Steven E. Petersen, Kevin J. Black, Bradley L. Schlaggar

**Affiliations:** ^1^ Department of PsychiatryWashington University School of MedicineUSA; ^2^ Department of RadiologyWashington University School of MedicineUSA; ^3^ Department of NeurologyWashington University School of MedicineUSA; ^4^ Department of NeuroscienceWashington University School of MedicineUSA; ^5^ Department of PediatricsWashington University School of MedicineUSA; ^6^ Department of PsychologyThe University of Texas at AustinUSA

## Abstract

Tourette syndrome (TS) is a developmental neuropsychiatric disorder characterized by motor and vocal tics. Individuals with TS would benefit greatly from advances in prediction of symptom timecourse and treatment effectiveness. As a first step, we applied a multivariate method – support vector machine (SVM) classification – to test whether patterns in brain network activity, measured with resting state functional connectivity (RSFC) MRI, could predict diagnostic group membership for individuals. RSFC data from 42 children with TS (8–15 yrs) and 42 unaffected controls (age, IQ, in‐scanner movement matched) were included. While univariate tests identified no significant group differences, SVM classified group membership with ~70% accuracy (*p* < .001). We also report a novel adaptation of SVM binary classification that, in addition to an overall accuracy rate for the SVM, provides a confidence measure for the accurate classification of each individual. Our results support the contention that multivariate methods can better capture the complexity of some brain disorders, and hold promise for predicting prognosis and treatment outcome for individuals with TS.

## Research highlights

- Support vector machine learning was used to classify children with and without TS based solely on resting state functional connectivity data.
- Patterns in functional connectivity can significantly discriminate between children with and without TS.
- Individual child classification demonstrated reliable accuracy for most children.

## Introduction

Tourette syndrome (TS) is a neuropsychiatric disorder with childhood onset, characterized by motor and vocal tics (Leckman, King & Bloch, 2014). Tics are brief, unwanted, repetitive movements or noises; common examples include forceful blinking, head jerking, sniffing, and throat clearing (Black, 2001). TS affects 1–3% of children and is more prevalent in boys than girls (~4:1) (Cubo, Gabriel y Galan, Villaverde, Velasco, Benito *et al*., 2011; Khalifa & von Knorring, 2003; Scahill, Bitsko & Visser, 2009). Typically, tic symptoms begin at about 6–7 years of age, peak in severity in early adolescence, and then improve into late adolescence and early adulthood (Leckman, Bloch, King & Scahill, 2006; Leckman, Riddle, Hardin, Ort, Swartz *et al*., 1998). An estimated 60% of children with TS also have ADHD and 30% also have OCD; in fact, only 10% of children with TS have no comorbid conditions at all (Freeman, Fast, Burd, Kerbeshian, Robertson *et al*., 2000). Thus, while tics are the defining symptom of TS, the condition involves multiple neuropsychiatric problems, all of which can impact quality of life (Cavanna, Schrag, Morley, Orth, Robertson *et al*., 2008; Eddy, Rizzo, Gulisano, Agodi, Barchitta *et al*., 2011; Stewart, Greene, Lessov‐Schlaggar, Church & Schlaggar, 2015).

As with many developmental neuropsychiatric disorders, TS is heterogeneous with respect to symptom severity, symptom progression, and clinical outcome. For example, an obtrusive tic such as shouting socially inappropriate words likely interferes with daily life more than an unobtrusive tic such as sniffing. In addition, while the developmental trajectory of tic symptoms described above is ‘typical’, many people with TS do not follow this developmental course. The syndrome as a whole can manifest differently in different individuals, and can change over time within a person. Further, high comorbidity rates (Freeman *et al*., 2000) and findings of atypical cognitive function (Cavanna, Eddy & Rickards, 2009) emphasize that TS involves more than just tics. Thus, there is a great need for the ability to predict future clinical course and responses to treatments at the individual level.

Since TS is a neuropsychiatric disorder, data from the brain may help to inform predictions of clinical outcome. However, research investigating the brain in TS is incomplete. Most studies have focused on restricted brain regions, with a particular emphasis on the motor system given that tics are the defining symptoms. For example, nonhuman primate studies have targeted the striatum, demonstrating that microinjection of bicuculline (a GABA‐A antagonist) into the putamen, effectively stimulating putamen neurons (through blocking inhibition), resulted in tic‐like movements (Bronfeld, Belelovsky & Bar‐Gad, 2011; McCairn, Bronfeld, Belelovsky & Bar‐Gad, 2009). In humans, postmortem studies focusing on the striatum have demonstrated reduced numbers of parvalbumin positive GABAergic interneurons in patients with TS (Kalanithi, Zheng, Kataoka, DiFiglia, Grantz *et al*., 2005; Kataoka, Kalanithi, Grantz, Schwartz, Saper *et al*., 2010). Neuroimaging studies that investigate humans *in vivo* have yielded largely inconsistent results (for a review, see Greene, Black & Schlaggar, 2013); however, the most consistent finding is reduced caudate volume in children and adults with TS (Hyde, Stacey, Coppola, Handel, Rickler *et al*., 1995; Müller‐Vahl, Kaufmann, Grosskreutz, Dengler, Emrich *et al*., 2009; Peterson, Riddle, Cohen, Katz, Smith *et al*., 1993; Peterson, Thomas, Kane, Scahill, Zhang *et al*., 2003). In addition to the striatum, human neuroimaging evidence has implicated cortical regions that support somatosensory and motor functions, including thinning in sensorimotor cortical regions (Sowell, Kan, Yoshii, Thompson, Bansal *et al*., 2008; Worbe, Gerardin, Hartmann, Valabregue, Chupin *et al*., 2010) and increased fMRI activity in the supplementary motor area prior to a tic (Bohlhalter, Goldfine, Matteson, Garraux, Hanakawa *et al*., 2006; Hampson, Tokoglu, King, Constable & Leckman, 2009). Thus, there is converging evidence for the involvement of somatomotor cortical and subcortical regions in TS. However, TS is quite complex, and these somatomotor results do not easily encompass all aspects of TS symptomatology.

The brain is a highly interconnected system composed of multiple functional networks (Power, Cohen, Nelson, Wig, Barnes *et al*., 2011), and TS is unlikely to be the result of atypical functioning limited to sensorimotor networks. One method used to study large‐scale whole brain systems that has become increasingly popular in recent years is resting state functional connectivity (RSFC) MRI. RSFC measures low‐frequency (< 0.1 Hz) spontaneous fluctuations in the blood oxygenation level dependent (BOLD) signal while the subject lies awake in the scanner during the absence of a task. By calculating correlations between the BOLD timeseries of different brain regions, this method can reveal functional brain systems that are both biologically plausible and replicable (Fox & Raichle, 2007). Thus, RSFC can be used to study large‐scale functional systems, and does not face the potential susceptibility to performance confounds that can pose problems for task fMRI experiments. Our laboratory previously used RSFC to study children and adolescents with TS (Church, Fair, Dosenbach, Cohen, Miezin *et al*., 2009a), focusing on two cognitive control systems in the brain: the frontoparietal system, which supports adaptive online task control, and the cingulo‐opercular system, which supports maintenance of task sets (Dosenbach, Fair, Cohen, Schlaggar & Petersen, 2008; Dosenbach, Visscher, Palmer, Miezin, Wenger *et al*., 2006). We found evidence for atypical and immature functional connectivity in these control systems, and found corroborating evidence for these findings with task fMRI (Church, Wenger, Dosenbach, Miezin, Petersen *et al*., 2009b). Others have found evidence for immature functional connectivity within cortico‐striatal systems in adults with TS using RSFC (Worbe, Malherbe, Hartmann, Pelegrini‐Issac, Messe *et al*., 2012) and atypical structural connectivity in cortico‐striatal pathways in adults with TS using diffusion‐weighted imaging and probabilistic tractography (Worbe, Marrakchi‐Kacem, Lecomte, Valabregue, Poupon *et al*., 2015). One study investigated the directionality of functional relationships between brain regions using effective connectivity methods, and demonstrated stronger cortico‐cortical and striato‐cortical connectivity in TS mapping onto sensorimotor systems (Wang, Maia, Marsh, Colibazzi, Gerber *et al*., 2011; for a comment, see Greene & Schlaggar, 2012). Thus, measures of connectivity have begun to reveal atypical function in multiple brain systems in TS.

The studies to date, however, have targeted a limited number of regions at a time, using traditional univariate analyses to test for group differences. Such analysis strategies are indeed useful for answering questions about targeted brain regions (e.g. caudate) or particular brain systems (e.g. the frontoparietal control system). They are limited, though, in that these methods require some a priori knowledge guiding the choice for the selected brain regions or systems of interest. TS research could benefit from studies that examine multiple systems covering the whole brain in an unbiased way, and that test the interactions between regions within and between those systems. Realistically, studying the whole brain at once can lead to statistical problems of multiple comparisons, in the face of which traditional univariate methods may not be sensitive enough to detect any true underlying group differences, especially with limited sample sizes. Such methodological challenges may explain the inconsistent results in the TS literature, particularly in the human neuroimaging literature (Greene *et al*., 2013; Greene, Schlaggar & Black, 2015).

One approach for studying whole brain data is support vector machine (SVM) classification. SVMs are machine‐learning algorithms that allow for diagnostic classification/prediction of individuals based on an underlying multivariate statistical analysis of the data. This method interrogates *patterns* of multidimensional data to discriminate classes of data points, such as diagnostic groups. Thus, SVMs consider data from all features (e.g. brain regions) in aggregate, as opposed to considering data from each feature as an individual entity as in traditional univariate statistical methods. SVMs can handle many types of data and high‐dimensional feature spaces, making them attractive for analyzing neuroimaging data. Given the many functional connections that can be examined with RSFC, SVM classification is a prime candidate for discriminating patient groups based on widespread differences in functional connectivity.

Our laboratory has previously demonstrated that SVM algorithms can predict group membership of children or adults with ~90% accuracy, showing (as a proof of principle) that RSFC can be used for highly‐accurate diagnostic classification (Dosenbach, Nardos, Cohen, Fair, Power *et al*., 2010). Since then, several groups have shown significant classification accuracies using RSFC data for a number of patient populations, including autism (Anderson, Nielsen, Froehlich, DuBray, Druzgal *et al*., 2011b; Uddin, Supekar, Lynch, Khouzam, Phillips *et al*., 2013), ADHD (Fair, Nigg, Iyer, Bathula, Mills *et al*., 2012), and schizophrenia (Fan, Liu, Wu, Hao, Liu *et al*., 2011). Yet, it should be noted that it is unclear whether or not these studies (with the exception of Fair *et al*.) accounted adequately for artifacts that are problematic in RSFC data, particularly head movement in the scanner. SVM classification methods are extraordinarily sensitive to differences between groups in the data, whether those differences are driven by true brain activity or noise. Thus, if a patient group moves in the scanner more than the comparison group (which is often the case), classification accuracy can be easily inflated and interpretations can be distorted.

Given that the goal of SVM classification is to make predictions about unknown samples, the method is well suited for the type of individualized predictions desirable for eventual clinical use. However, before we can predict clinical course or treatment outcome for an individual child, we must first demonstrate prediction ability. Therefore, critically, we tested whether brain activity alone could accurately predict diagnosis for children with and without TS. While this binary classification approach does not account for the complex clinical heterogeneity of TS, this first step is necessary: if brain imaging data can be used to make predictions about individual children even at the level of binary diagnostic classification, then such results hold promise for further refinement of the methods to ultimately predict prognosis and treatment responses. Perhaps the same information used to classify individuals by diagnosis can then be used to predict future outcomes.

In the present study we used RSFC to test whether functional connectivity among many brain systems is a useful biomarker of diagnostic status. We aimed to test a group of children with TS who represented well the true population of TS. Thus, we did not exclude for comorbid conditions or current medication use, given that most children with TS have comorbid conditions and those in need of clinical care often take medications. This naturalistic data collection approach helps maintain generalizability to the majority of TS patients and avoids ignoring those patients who most often seek clinical care (Gilbert & Buncher, 2005; Greene, Black & Schlaggar, in press). Using RSFC data from these children, we implemented SVM classification to test whether functional connectivity across much of the brain contains sufficient discriminating information to predict diagnostic status for an individual child (i.e. to predict whether or not the child has TS). Based on previous demonstrations of group membership classification using RSFC, we hypothesized that RSFC data covering much of the brain would carry information that can discriminate children with TS and control children.

## Materials and methods

### Participants

Children with TS were recruited from the Washington University School of Medicine Movement Disorder Center and the Greater Missouri chapter of the Tourette Syndrome Association. Tic‐free controls were recruited from the Washington University campus and the surrounding community. Conditions commonly comorbid with TS (ADHD, OCD, anxiety) and medication use were not considered exclusionary for the TS group, but were for the control group. All participants were native English speakers. All participants underwent a two‐scale brief assessment of IQ (WASI). For TS participants, the experimenter (JAC, KJB) completed the following measures of ‘past week’ symptom severity: Yale Global Tic Severity Scale (YGTSS; Leckman, Riddle, Hardin, Ort, Swartz *et al*., 1989), Children's Yale‐Brown Obsessive Compulsive Scale (CY‐BOCS; Scahill, Riddle, McSwiggin‐Hardin, Ort, King *et al*., 1997), and ADHD Rating Scale (Conners, Sitarenios, Parker & Epstein, 1998).

A total of 83 children with TS participated in the study procedures. To minimize the effects of head movement on RSFC data, only those participants with a minimum of 125 frames (5.2 minutes) of RSFC data remaining after motion ‘scrubbing’ (see below) were included, yielding usable data from 42 children with TS (8–15 years old, mean age 12.3; 34 males). A group of 42 tic‐free controls (8–15 years old, mean age 12.1; 34 males), also with a minimum of 125 frames of usable RSFC data, and matched to the TS group on age, sex, IQ, handedness, and in‐scanner movement, were selected from an extant database of participants (*n* = 487, ages 6.7–35.4 years old, 206 males). Control participants reported no history of neurological or psychiatric disease and were not on psychotropic medications, as assessed by parental report. A parent or guardian for all child participants gave informed consent and all children assented. All participants were compensated for their participation. The Washington University Human Research Protection Office approved all studies.

### Image acquisition

Data were acquired on a Siemens 3T MAGNETOM Trio scanner (Erlanger, Germany) with a Siemens 12‐channel Head Matrix Coil. Each child was fitted with a thermoplastic mask fastened to the head coil to help stabilize head position. T1‐weighted sagittal MP‐RAGE structural images (one sequence acquisition for each control participant and for each of 29 TS participants: slice time echo = 3.06 ms, TR = 2.4 s, inversion time = 1 s, flip angle = 8°, 176 slices, 1 × 1 × 1 mm voxels; two sequence acquisitions for each of the 13 remaining TS participants: slice time echo = 2.34 ms, TR = 2.2 s, inversion time = 1 s, flip angle = 7°, 160 slices, 1 × 1 × 1 mm voxels) and a T2‐weighted turbo spin echo structural image (TE = 84 ms, TR = 6.8 s, 32 slices, 2 × 1 × 4 mm voxels) in the same anatomical plane as the BOLD images were obtained to improve alignment to an atlas. Functional images were acquired using a BOLD contrast‐sensitive echo‐planar sequence (TE = 27 ms, flip angle = 90°, in‐plane resolution 4 × 4 mm; volume TR = 2.5 s). Whole‐brain coverage was obtained with 32 contiguous interleaved 4 mm axial slices. Steady‐state magnetization was assumed after four volumes. For most participants (40 TS, 34 controls), 2–4 resting state sequences lasting 5–5.5 min each were acquired. The remaining two TS participants completed three sequences lasting 6.8 minutes each; of the seven remaining control participants, four of them completed four sequences lasting 3.2 minutes each and three of them completed one sequence lasting 30 minutes. In the TS group, 388 ± 61.5 (range 264–528) total functional volumes were acquired, and in the control group, 372 ± 130 (range 260–724) total functional volumes were acquired.

During functional ‘resting’ scans, participants viewed a centrally presented white crosshair (subtending <1° visual angle) on a black background. Participants were instructed to relax, ‘keep an eye on the plus sign’, and hold as still as possible.

### Image analysis

#### Preprocessing

Data from all participants underwent the same processing steps. First, functional images from each participant were preprocessed to reduce artifacts (Shulman, Pope, Astafiev, McAvoy, Snyder *et al*., 2010). These steps included: (i) sinc interpolation of all slices to the temporal midpoint of the first slice, accounting for differences in the acquisition time of each individual slice, (ii) correction for head movement within and across runs, and (iii) intensity normalization to a whole brain mode value (across voxels and TRs) of 1000 for each run. Atlas transformation of the functional data was computed for each individual via the T2‐weighted and MP‐RAGE T1‐weighted scans (using the average of two MP‐RAGE scans for those participants with two MP‐RAGE scans). Each run was then resampled in atlas space on an isotropic 3 mm grid combining movement correction and atlas transformation in a single interpolation (Shulman *et al*., 2010). The target atlas (TRIO_KY_NDC) was created using validated methods (Black, Koller, Snyder & Perlmutter, 2004) from MP‐RAGE scans from 13 7‐ to 9‐year‐old children (seven males) and 12 21‐ to 30‐year‐old adults (six males). Construction of this atlas consisted of a bootstrap method to register the individual MP‐RAGE scans to atlas space followed by an iterative refinement method of the average atlas image. This target atlas strategy minimizes systematic age differences in the atlas location of cortical structures (Burgund, Kang, Kelly, Buckner, Snyder *et al*., 2002). The scans for the atlas were collected on the same Siemens 3T MAGNETOM Trio used in the present study. The atlas was made to conform to the Talairach atlas space as defined by the SN method of Lancaster, Glass, Lankipalli, Downs, Mayberg *et al*. (1995).

#### Functional connectivity preprocessing

Several additional preprocessing steps were applied to all participant data to reduce spurious variance unlikely to reflect neuronal activity (Fox, Zhang, Snyder & Raichle, 2009). These RSFC preprocessing steps included: (i) demeaning and detrending, (ii) multiple regression of nuisance variables from the BOLD data, (iii) temporal band‐pass filtering (0.009 Hz < *f *< 0.08 Hz), and (iv) spatial smoothing (6 mm full width at half maximum). Nuisance variables included motion regressors derived by Volterra expansion (Friston, Williams, Howard, Frackowiak & Turner, 1996), individualized ventricular and white matter signals, brain signal averaged across the whole brain, and the derivatives of these signals.

Since head motion can cause spurious yet systematic changes in BOLD correlations, which affect group comparisons (Power, Barnes, Snyder, Schlaggar & Petersen, 2012; Van Dijk, Sabuncu & Buckner, 2012), we implemented a volume censoring (motion ‘scrubbing’) procedure as in Power, Mitra, Laumann, Snyder, Schlaggar *et al*. (2014) to minimize motion‐related effects. Frame‐by‐frame head displacement (FD) was calculated from preprocessing realignment estimates, and volumes with FD > 0.2 mm were flagged for censoring. Volumes were included (not censored) only in temporally contiguous sets of at least five volumes with FD < 0.2 mm each, and BOLD runs were included only if they retained a minimum of 30 such volumes. In order to equate the number of volumes across participants after volume censoring, the first 125 volumes of usable RSFC data (i.e. volumes surviving ‘scrubbing’ procedures) for each participant were submitted to further analyses.

The flagged data were then run through RSFC preprocessing. First, the following steps were applied to the censored data: (i) demeaning and detrending, and (ii) multiple regression of nuisance variables. Then the censored frames were replaced with interpolated data, using least squares spectral estimation, and the band‐pass filter and spatial smoothing were run again on the now ‘uncontaminated’, interpolated data, as in Power *et al*. (2014). The interpolated data were then re‐censored for final analyses.

#### Functional connectivity network construction

For each participant, the RSFC timecourse was extracted from a previously defined set of 264 regions of interest (ROIs) covering much of the brain (Power *et al*., 2011). These 264 ROIs can be organized into separable brain systems (e.g. default‐mode, fronto‐parietal, visual, etc.). ROIs were modeled as 10 mm spheres. A correlation matrix was formed by calculating the correlation of each ROI's timecourse with every other ROI's timecourse, resulting in a 264 × 264 correlation matrix for each subject. The correlations were then normalized using Fisher's *r*‐to‐*z* transform. Correlation matrices were then concatenated across all *n* subjects to form a 264 × 264 × *n* matrix, which was used for subsequent analyses.

#### Univariate *t*‐tests

We first compared the TS group and the control group using traditional univariate methods. Independent‐samples *t*‐tests (assuming unequal variance) were performed on all 34,584 functional connections represented in the 264 × 264 correlation matrices. Connections with |*r*| < 0.1 in both groups were not included in the analyses. False Discovery Rate correction was applied to account for multiple comparisons.

#### Support vector machine (SVM) classification

SVM algorithms were used to test how well RSFC data could classify an individual child's diagnostic status (i.e. TS or control). We used the Spider Machine Learning Toolbox implemented in Matlab (http://www.kyb.mpg.de/bs/people/spider/) for SVM computations and applied methods previously used in our lab (Dosenbach *et al*., 2010; Greene, Laumann, Dubis, Ihnen, Neta *et al*., 2014).

Briefly, a training set of labeled samples, each with a series of features, is supplied to the SVM. In our analyses, the training set was composed of the 264 ROIs × 264 ROIs × 84 participants correlation matrix; each participant was treated as a labeled sample (TS or control) and each of the 34,584 functional connections (*r* values) was treated as a feature. The algorithm then learns the properties and weights of the features that characterize the labels. Once a decision function is learned based on the training data, it can be used to predict the class label of a new, previously unseen, test sample (Ben‐Hur, Ong, Sonnenburg, Schölkopf & Rätsch, 2008; Schölkopf & J. Smola, 2002). For our analyses, we implemented linear SVM. Using soft‐margin separation (Ben‐Hur *et al*., 2008), linear kernel SVMs were applied to distinguish between children belonging to two classes: TS and control.

To estimate classification accuracy, we used a leave‐one‐out‐cross‐validation (LOOCV) method, where each participant was designated as the test sample in turn, while the remaining samples were used to train the SVM classifier. As each participant was designated as the test sample once, there were as many folds (rounds of cross‐validation) as participants. Within each LOOCV fold, feature reduction was applied, as this technique has been shown to speed up computation and improve classification performance (De Martino, Valente, Staeren, Ashburner, Goebel *et al*., 2008; Hastie, Tibshirani & Friedman, 2001; Pereira, Mitchell & Botvinick, 2009). As in Dosenbach *et al*. (2010), two‐sample *t‐*tests (not assuming equal variance) were computed to compare each feature across the two classes on the training set of each LOOCV fold (with the test sample excluded). The features with the highest *t* scores were retained. Once all folds were completed, the accuracies for all folds were averaged together to generate a final accuracy estimate (percent of participants accurately classified), and the one‐tailed probability of the accuracy estimate (under the null hypothesis that each participant assignment was random with probability 0.5) was calculated using the binomial distribution. To identify the features that most contributed to the classification, those features that were retained in all LOOCV folds (100% consensus) for each SVM were extracted.

For feature reduction, previous studies have chosen the number of features to test a priori (e.g. 200 features was chosen in Dosenbach *et al*., 2010). It is possible that choosing a particular number of features may result in spuriously high or low classification accuracy. Therefore, we tested multiple feature numbers, specifically 100, 200, 300, 400, and 500 features, in order to view the stability of the classification accuracy.

Using the 264 ROIs, we also ran an experimental control SVM. Rather than dividing the 84 children into two groups according to diagnosis (TS vs. control), we pseudo‐randomized group membership such that each group of 42 included 21 TS and 21 control participants such that the groups of 42 were matched on age, sex, IQ and movement. Thus, our experimental control SVM aimed to classify Group 1 (21 TS + 21 controls) vs. Group 2 (21 TS + 21 controls). In addition, we ran permutation testing in which the group labels were randomized for 1000 permutations. Given that the peak accuracy for classifying diagnostic status was obtained when using 400 features, the permutation testing was run using 400 features.

We ran additional SVM analyses to assess the diagnostic relevance of specific brain systems in a targeted fashion. We generated correlation matrices that included only those ROIs belonging to particular brain systems thought to be involved in TS. Specifically, we investigated somatomotor regions, as TS is a movement disorder, and control systems (frontoparietal, cingulo‐opercular, salience, dorsal attention, and ventral attention systems), given previous reports of atypical cognitive control in TS. We investigated the somatomotor ROIs and control systems in separate and in joint SVM analyses. We also investigated ‘processing’ systems (visual, auditory, somatomotor) to test whether these primary cortical regions, without association regions, could distinguish the groups. Based on previous work implicating motor circuitry and cognitive control systems in TS, we expected that the SVMs including such systems would yield higher classification accuracy than the SVM that includes only processing systems.

#### Individual classification accuracy

Since the primary goal is diagnostic classification for individuals, we investigated a novel adaptation of SVM binary classification to measure ‘individual accuracy’, i.e. the rate at which an individual is classified accurately, for each of our 84 participants. We used the parameters of the SVM classifier with the highest classification accuracy for the group (which was the analysis of all 264 ROIs and 400 features, as described in the Results). Specifically, a leave‐two‐out‐cross‐validation method was implemented, in which one participant was removed as the test case of interest while the remaining participants underwent the LOOCV procedure. Each LOOCV resulted in *n*−1 models (LOOCV folds). Then, the test case of interest was tested on each of those models, and an average individual accuracy was calculated for that case. This procedure was repeated for every participant. This individual accuracy reflects how reliably that participant is classified across models. Thus, we moved a step beyond classification alone and developed a measure of classification confidence.

#### Support vector regression (SVR)

We also tested whether RSFC data could predict a continuous measure of symptom severity in the TS group using SVR. By extending SVM to predict a continuous variable, specifically YGTSS Total Tic score and YGTSS Motor Tic score (several children did not have vocal tics during the past week), instead of a binary class label (TS/control), we tested whether patterns of functional connectivity could predict tic severity for individuals. We applied SVR methods as in Dosenbach *et al*. (2010).

Finally, we sought to determine whether differences between TS and controls were more consistent with immature functional connectivity or atypical functional connectivity. We used SVR to test whether any differences in the RSFC of TS participants reflect typical, yet immature development. We used RSFC data from a developmental sample of 129 healthy participants (7–31 years old; mean age 16.6; 60 females; 33 participants overlapped with the 42 control participants described in ‘Participants’ and in Table 1) in which image acquisition, preprocessing, and functional connectivity network construction were accomplished using the same methods as described above. First, we applied SVR to the healthy developmental group using a LOOCV approach to determine *n* (i.e. 129) models able to predict an individual's maturity given the individual's RSFC. For feature reduction, we tested the SVR at 200, 400, 600, 800, and 1000 features because SVRs can require more features for accurate prediction than SVMs; 1000 features best captured the developmental trajectory. Second, we tested each of the 42 TS participants on the 129 derived developmental SVR models to determine where these children fell on the functional connectivity maturation curve relative to their chronological age.

**Table 1:** Participant characteristics shown as mean (SD); range

	TS group	Control group
*N*	42	42
Male/Female	34/8	34/8
Age (years)	12.3 (2.2); 8.1–15.7	12.1 (2.0); 8.7–15.3
IQ	111.4 (12.8); 87–135	112 (12.9); 86–136
In‐scanner movement (FD)	0.102 (0.001); 0.007–0.13	0.098 (0.02); 0.06–0.13
YGTSS Total Tic Score	16.5 (8.0); 0–39	NA
ADHD Rating	11.5 (9.3); 0–34	NA
CY‐BOCS Score	5.9 (7.5); 0–27	NA
Number on medications	20	0
Number with diagnosed comorbidities	25	0

Note

FD = framewise displacement, measured in millimeters (mm).

Control participants did not complete the YGTSS, ADHD Rating Scale, or CY‐BOCS.

## Results

### Participants

Participant characteristics for the 42 TS and 42 control participants are summarized in Table 1. Individual participant information is provided in Table S1. There were no significant differences between groups in age, sex, IQ, or in‐scanner movement after motion ‘scrubbing’ procedures, all *p*s > .1. Twenty‐five TS participants reported diagnosed comorbid conditions, most commonly ADHD and OCD, and 20 reported taking psychoactive medications at the time of the scan, mostly centrally acting adrenergic agents and stimulants (Table 2). Given that a total of 83 children with TS participated in the study and 41 were excluded due to motion, we tested whether the final sample of 42 children with TS was biased toward those with less severe symptoms. We found no difference in symptom severity (YGTSS Total Tic score) between the 42 children included in the final sample (*M* = 16.5, *SD* = 8.6, range 0–39) and the 41 children excluded due to motion (*M* = 17.2, *SD* = 8, range = 0–34), *t*(79)  = 0.38, *p* = .70.

**Table 2:** Profile of medication use for the 20 medicated TS participants at time of scan

Medication class	Number of participants
Centrally‐acting adrenergic agents	14
Stimulants	8
Antihistamines	2
SSRI antidepressants	1
Tricyclic antidepressants	1
Atypical Neuroleptics	1
Norepinephrine RI for ADHD	1
β blockers	1
Corticosteroids	1
Sulfonamide	1
Hypnotics	1

Note

13 TS participants were taking more than one medication.

### Univariate analyses failed to show reliable group differences in functional connectivity

**Figure 1:**
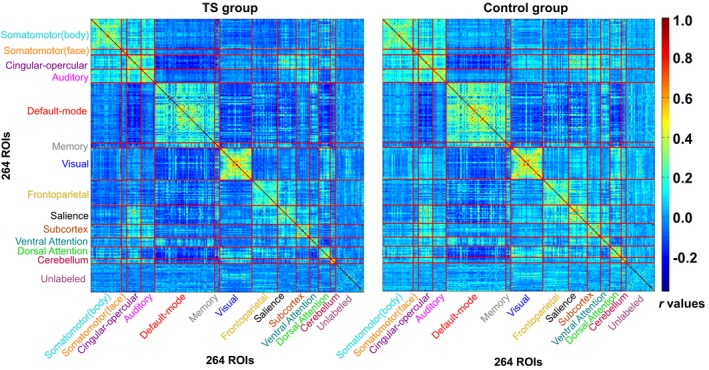
Group average 264 ROI × 264 ROI correlation matrices show the expected network block structure. ROIs are organized by the brain system to which they belong; red lines demarcate boundaries between systems. *r* values are Fisher‐*Z* transformed.

**Figure 2:**
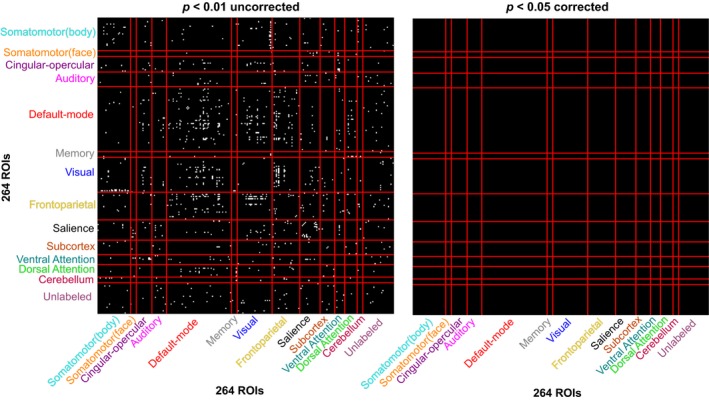
Independent‐samples *t*‐test results comparing TS and control groups on functional connections between 264 ROIs. No functional connections survived FDR correction for multiple comparisons. Significant connections denoted in white; 264 ROIs are organized by the brain system to which they belong; red lines demarcate boundaries between systems.

Figure 1 shows the group averaged correlation matrices for the 42 TS and 42 control participants. Qualitatively, the groups look similar. To quantify potential differences between groups, independent samples *t*‐tests were run on the subject correlation matrices. As shown in Figure 2, no connections were significantly different after FDR correction.

### SVM classification distinguished TS and controls based on functional connectivity

**Figure 3:**
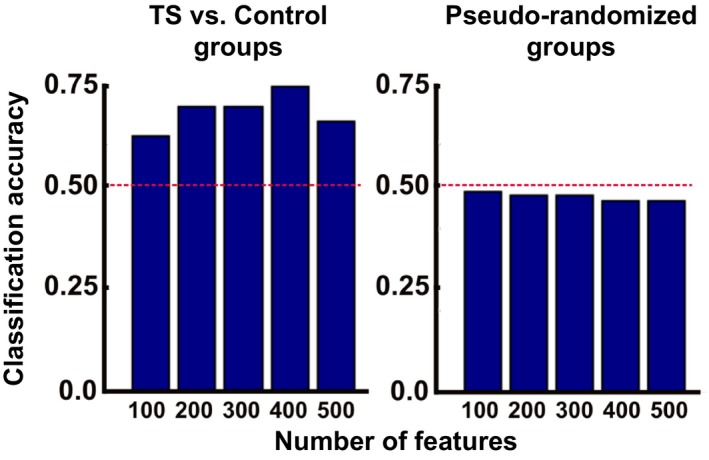
Classification accuracy for SVMs using 264 ROIs after feature reduction to 100–500 features. Left: TS group (*n* = 42) vs. age‐, sex‐, IQ‐ and movement‐matched control group (*n* = 42) classifier. Right: Pseudo‐randomized groups (*n* = 21 TS and 21 controls in each group) classifier. Dotted red line denotes chance accuracy.

Using the full set of 264 ROIs, the accuracy of classifying a child as TS or control was reasonably stable around 70% (Figure 3). The peak accuracy was 74% (p < .001), with sensitivity of 76% and specificity of 71%, using 400 features. Thus, resting state fMRI data can be used to classify an individual child as having TS or not with significant accuracy. The experimental ‘control’ SVM, in which the group labels were pseudo‐randomized such that each group of 42 contained 21 children from the TS group and 21 from the control group, resulted in classification accuracy at chance levels (46–48%). Further, the permutation testing demonstrated that the 74% accuracy obtained when using 400 features was on the tail of the distribution (*p* = .004, Figure S1). Thus, the ~70% classification accuracy for distinguishing TS and controls was not likely due to chance.

**Figure 4:**
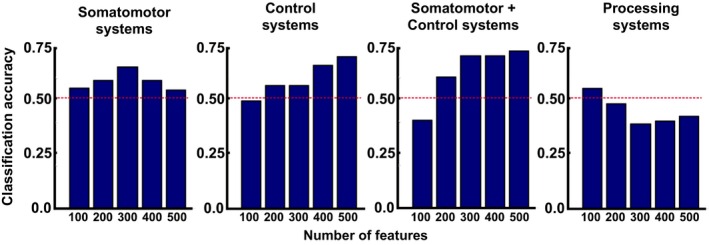
Classification accuracy for SVMs restricted to ROIs belonging to targeted brain systems after feature reduction to 100–500 features. Dotted red line denotes chance accuracy. Somatomotor = somatomotor body and somatomotor face systems; Control = frontoparietal, cingulo‐opercular, salience, dorsal attention, and ventral attention systems; Processing = visual, auditory, somatomotor body, and somatomotor face systems.

Classification accuracies were similarly computed for additional SVMs that targeted selective brain systems. As shown in Figure 4, the SVM that targeted somatomotor regions and control systems together yielded accuracies higher than the SVMs that targeted somatomotor regions or control systems alone, and the SVM that targeted only processing systems was near chance accuracy. Notably, classification accuracy for the SVM that included both somatomotor regions and control systems was closest to that of the entire 264‐region SVM (peak accuracy 73%, using 500 features).

### Features driving the classification involved many brain systems

To interrogate the functional connections driving the distinctions between TS and controls, we examined the features for the SVM with the highest classification accuracy (i.e. 264 regions, 400 features, classification accuracy = 74%). For this SVM, we examined the 100% consensus features, that is, those features retained during the feature reduction step for every LOOCV fold. This process resulted in the identification of 249 consensus features. Since SVM allows for the extraction of feature weights, providing information regarding the most heavily weighted features that contribute to the classification, we extracted the feature weights for the consensus features. Examination of the specific functional connections revealed connections spanning many of the defined brain systems (Figure S2).

### Individual participant classification demonstrated reliable accuracy for most participants

**Figure 5:**
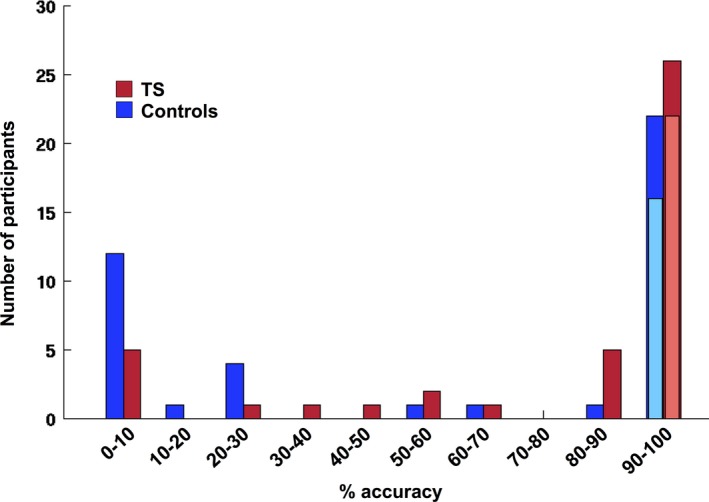
Individual participant classification shows that most participants are classified accurately most of the time. Lighter shades of blue and red denote participants with 100% classification accuracy.

Results from the leave‐two‐out‐cross‐validation method used to measure classification confidence for individual participants are shown in Figure 5. Most participants (64%) had individual accuracy greater than 80%, and 45% of all participants were classified accurately in every model (100% individual accuracy). Twelve children in the control group and five children in the TS group were almost always misclassified (individual accuracy < 10%), so we examined the characteristics of these participants (see Table S1). The five consistently misclassified TS participants were of varying ages and IQs, one was female, one was left‐handed, and they included children with and without comorbid diagnoses as well as children on and off medications. The 12 consistently misclassified control participants were also of varying ages and IQs, two were female, and two were left‐handed. Thus, overall we did not find a systematic explanation.

In order to test whether or not classification accuracy was influenced by medication status, we compared individual participant data between medicated and unmedicated TS participants. There was no significant difference in individual accuracy between the medicated (*M* = 74.3%, *SD* = 37.1) and unmedicated (*M* = 83.3%, *SD* = 30.9) TS participants, *t*(40) = 0.86, *p* = .4. There was also no significant difference in the proportion of TS children classified correctly (defined by individual accuracy > 60%) who were medicated (16/22) and unmedicated (16/20), χ^2^ (1, *N* = 42) = 0.31, *p* = .58. Since the most common comorbid condition was ADHD, we performed similar analyses to compare those TS participants with and without ADHD (NB: these analyses excluded five participants who did not fit cleanly into either the ‘with ADHD’ or ‘without ADHD’ groups: three with other comorbid diagnoses but no ADHD, one with past ADHD, and one with sub‐threshold ADHD). There was no significant difference in individual accuracy between TS participants with ADHD (*M* = 84.7%, *SD* = 25.1) and without ADHD (*M* = 74.4%, *SD* = 40.5), *t*(26) = 0.91, *p* = .37, and no significant difference in the proportion of TS children classified correctly (individual accuracy > 60%) with ADHD (16/20) and without ADHD (13/17), χ^2^ (1, *N* = 37) = 0.07, *p* = .79. These results suggest that classification accuracy was not driven by medication status or comorbid ADHD.

### SVR did not predict clinical tic severity

SVR was unable to successfully predict YGTSS Total Tic scores (*r* ^*2*^ = .036, *p* = .23), perhaps because the predicted scores fell within a restricted window (10–19) of the actual range of scores (0–39). Similarly, SVR was unable to predict YGTSS Motor Tic score (*r* ^*2*^ = .015, *p* = .44). Thus, RSFC may not carry information about tic severity, past week YGTSS score may be a suboptimal measure of tic severity at the moment of the scan (as it assesses tic during the ‘past week’), or we may not have adequate power to predict severity.

### SVR predicted chronological age for children with and without TS comparably

We also applied a developmental SVR, first reproducing our previous functional brain maturity prediction results in typical development (Dosenbach *et al*., 2010). In a healthy developmental sample (*n* = 129; 33 of whom overlapped with the 42 control participants), we demonstrated a maturation curve of predicted vs. chronological age using RSFC data across 264 regions and 1000 features, *r* ^2^ = 0.48 *p* < .001. When we then tested age predictions for the 42 children with TS using these typical development SVR models, the predicted age for the children with TS fell well within the typical development maturation curve (Figure S3). Thus, SVR predicted age for the children with TS similarly to typically developing children, demonstrating no evidence for functional immaturity.

## Discussion

### Patterns of functional connectivity can be used to classify children with and without TS with ~70% accuracy

The ability to predict symptom progression, clinical outcome, and response to treatment for individual patients would immensely benefit clinical care for TS and for other neuropsychiatric disorders as well. In the present study, we took a first step toward individualized predictions by testing whether an easily acquirable biological measure, RSFC, could be used to classify diagnostic status for children with TS. We found that RSFC data can classify children as having TS or not with ~70% accuracy, on average. We also investigated confidence levels for classifying each individual, finding that 64% of children had 80–100% individual accuracy. Traditional univariate analysis methods identified no significant differences in functional connectivity between children with TS and unaffected controls, yet SVM classification methods significantly discriminated the groups. Thus, there are patterns of functional connectivity that better distinguish children with and without TS than single functional connections. These results hold promise for SVM methods applied to TS, and with further refinement, are encouraging for future pursuits to predict clinical course and treatment.

Using an approach similar to that in this manuscript, our laboratory previously demonstrated classification of maturation status, classifying 7–11‐year‐olds and 24–30‐year‐olds as either children or adults, with 91% accuracy using RSFC data from 160 regions spanning multiple brain systems (Dosenbach *et al*., 2010) and with 75–83% accuracy using subcortical‐cortical RSFC (Greene *et al*., 2014). Other groups have also shown similar accuracies for age classification using RSFC data (Meier, Desphande, Vergun, Nair, Song *et al*., 2012; Supekar, Musen & Menon, 2009). Here, we demonstrate classification of diagnostic status for children with and without TS with ~70% accuracy. While this accuracy is not as high as our previous age‐group classification using very similar methods, one might predict that children with TS are more similar to other children than children are similar to adults. In fact, our results for TS classification are in line with previous reports of diagnostic classification for other neuropsychiatric disorders using RSFC data. These studies have shown accuracies ranging ~60‐90% (for a review, see Sundermann, Herr, Schwindt & Pfleiderer, 2014). Interestingly, those studies demonstrating accuracies in the upper part of that range predominantly classified adult patients with Alzheimer's disease and with schizophrenia from control participants (Chen, Ward, Xie, Li, Wu *et al*., 2011; Du, Calhoun, Li, Ma, Eichele *et al*., 2012; Fan *et al*., 2011; Koch, Teipel, Mueller, Benninghoff, Wagner *et al*., 2012; Shi, Liu, Jiang, Zhou, Zhu *et al*., 2007; Tang, Wang, Cao & Tan, 2012). The studies of childhood disorders, namely ADHD and autism, mostly demonstrated classification accuracies that were significant, yet in the lower part of the range (Anderson *et al*., 2011b; Bohland, Saperstein, Pereira, Rapin & Grady, 2012; Brown, Sidhu, Greiner, Asgarian, Bastani *et al*., 2012; Cheng, Ji, Zhang & Feng, 2012; Dai, Wang, Hua & He, 2012; Fair *et al*., 2012; Sato, Hoexter, Fujita & Rohde, 2012; Uddin *et al*., 2013). It is possible that there are more widespread RSFC aberrations in schizophrenia and Alzheimer's disease than in ADHD, autism, and TS, or more generally, in disorders of adulthood compared to those of childhood. Alternatively, some studies reporting higher classification accuracies could reflect a failure to account adequately for movement confounds, as group differences in sub‐millimeter head movements during fMRI data acquisition can lead to systematic changes in RSFC that masquerade as, or falsely enhance, group differences in functional architecture (Power *et al*., 2012). Thus, it is difficult to determine whether the higher classification accuracies previously reported for other diagnostic groups reflect a true or inflated discriminability of their groups of interest. In the present study, we implemented the most conservative current approaches in the field to minimize motion artifact (Power *et al*., 2014).

Here, we went a step beyond most previous studies of diagnostic classification, and investigated the reliability of each individual child's classification. Using an innovative leave‐two‐out‐cross‐validation approach, we demonstrated that 64% of all participants could be classified quite reliably (> 80% individual accuracy). In fact, 45% of the participants were classified correctly 100% of the time (100% individual accuracy). These results highlight the strength of SVM as a method for diagnostic prediction, as only 8% of individuals were classified near chance levels (50% individual accuracy). Interestingly, 17 of the 84 participants (5 TS, 12 controls) were consistently classified inaccurately, and we could not determine systematic characteristics that set them apart. Thus, some children with TS had functional connectivity organization more similar to their unaffected peers than to their fellow TS peers, while some unaffected children had functional connectivity organization more similar to children with TS. As more controls were consistently misclassified than children with TS, it is possible that some of the typically developing children had subclinical symptoms, undiagnosed past tics, or would later go on to develop symptoms. In fact, in our laboratory's most recent efforts to recruit typically developing children (data not included here), 15% of children who were deemed eligible after phone screening were later excluded due to current or past tics that were discovered only after in‐depth clinical assessment with both child and guardian. Indeed, classroom observation studies find that as many as 25% of children have tics at some point during childhood (Cubo *et al*., 2011; Snider, Seligman, Ketchen, Levitt, Bates *et al*., 2002), yet only 1–3% of children are diagnosed with a chronic tic disorder (Khalifa & von Knorring, 2003; Scahill, Specht & Page, 2014). Thus, there are many cases of undiagnosed and likely unrecognized tics.

### Multivariate methods may be more sensitive than univariate methods to detect group differences

The results from the present study demonstrate the power of using multivariate analysis approaches to discriminate individuals with a complex neuropsychiatric disorder from their unaffected peers. We investigated functional connectivity among a large number of regions, 264, in order to study functional connectivity across many brain systems in TS. First, we implemented traditional univariate analyses, which treat individual features (e.g. functional connections) separately, to ask in a hypothesis‐driven manner whether or not there is a significant group difference in a particular feature. Such analyses failed to identify significant differences in functional connectivity between children with and without TS after multiple comparisons correction. By contrast, SVM classification implements multivariate pattern analysis to find patterns in the data that reliably distinguish groups. We found that SVM algorithms were able to discriminate children with and without TS with significant classification accuracy. Thus, multivariate analyses may be more sensitive to detect abnormal patterns of brain activity in high‐dimensional data than traditional univariate tests (Jimura & Poldrack, 2012), supporting the contention that multivariate methods may be necessary to capture the complexity of some brain disorders (Bray, Chang & Hoeft, 2009).

### Interrogating the imaging features driving the classification can provide clues about brain connectivity differences between children with and without TS

While SVM methods, as used in this study, cannot identify specific biological mechanisms underlying TS, we took two approaches to examine the imaging features involved. Such examination can provide clues about those features that discriminate children with and without TS, informing future hypotheses about underlying biological mechanisms. Our primary analyses used a previously identified set of 264 regions that can be organized, using data‐driven community detection methods, into multiple functional brain systems (Power *et al*., 2011). Including the full set of 264 regions resulted in the highest and most stable classification accuracy. We interrogated the features driving the classification, and found functional connections involving numerous identified brain systems and spanning the entirety of the cerebral cortex. Visual examination of the features (Figure S2) did not reveal an obvious pattern, but showed the involvement of complex functional connections within and between many brain systems. As TS is a highly complex neuropsychiatric disorder, it is not necessarily surprising that the features driving the classification do not present as a neat, clear pattern. Thus, these findings provide new, not previously hypothesized, information that could only emerge when using an inclusive, multivariate approach.

We ran additional analyses in which we limited the regions included in the SVMs to targeted brain systems. Limiting the regions to somatomotor and higher‐level control systems yielded classification accuracy closest to that of the 264‐region classifier. Further, including somatomotor and control systems together yielded higher accuracy than including either group of systems alone (though still not higher than the 264‐region classifier), suggesting that the functional connections within *and* between these systems are important in discriminating TS from tic‐free controls. This targeted approach demonstrates construct validity, as limiting the analysis to systems thought to be involved in TS (somatomotor, control systems) yielded better accuracy than analysis limited to other systems (processing systems). Of course, the targeted classifiers still did not perform as well as the full 264‐region classifier, indicating that there is additional information in more widespread functional connectivity patterns that discriminate the groups. Again, since the approaches used here only provide clues about the spatial features involved, specific hypotheses about the biological mechanisms that may underlie these differences in connectivity patterns will need to be tested (e.g. studying pharmacological effects on classification accuracy) in order to pinpoint the specific mechanisms underlying TS.

The control systems examined here consisted of the fronto‐parietal, cingulo‐opercular, salience, dorsal attention, and ventral attention systems. The fronto‐parietal system, which includes the dorsolateral prefrontal cortex, inferior parietal cortex, and precuneus, is posited to support moment‐to‐moment adaptive task control, while the cingulo‐opercular system, which includes the anterior insula, dorsal anterior cingulate cortex, and thalamus, is thought to support stable task‐set maintenance (Dosenbach *et al*., 2008). The salience system also involves the anterior insula and dorsal anterior cingulate, yet the precise regions are separable from those of the cingulo‐opercular system (Power *et al*., 2011). The salience system has been described as an integrator of internal and external stimuli in order to facilitate adaptive behavior (Seeley, Menon, Schatzberg, Keller, Glover *et al*., 2007). The dorsal attention system comprises the putative frontal eye fields and dorsal parietal cortex, and supports goal‐driven attention, while the ventral attention system comprises the ventral frontal cortex and temporoparietal junction, and supports stimulus‐driven reorienting of attention (Corbetta, Patel & Shulman, 2008). While these control systems are dissociable, their interactions are important for cognitive and attentional control (Corbetta *et al*., 2008). Given the successful classification achieved when targeting these control systems, future studies investigating functional connectivity within and between these systems may illuminate the specific roles they play in TS.

TS is typically contextualized with other movement disorders. Consequently, research investigating the brain in TS has largely focused on the striatum and somatomotor cortical regions. The present results, however, support the involvement of other brain systems. We previously showed that systems supporting task control are involved in TS (Church *et al*., 2009a; Church *et al*., 2009b). Here, we demonstrate that patterns of functional connectivity involving many brain systems best discriminated patients with TS from their unaffected peers. Thus, TS likely involves more than just motor systems, and a broader conceptual view of TS is needed in order to advance our understanding of the underlying pathophysiology.

It is important to note that although our analyses consisted of binary classification of children as TS or control, we do not mean to suggest that TS is a single phenotype resulting from a single biological mechanism. Rather, just as our TS group is clinically heterogeneous, yet all have tics, we suspect that there may be multiple underlying mechanisms that share some RSFC features that link (most) individuals with TS together. These overlapping features can be detected by SVMs, making it possible to separate TS children from controls. Further refinement of the methods to account for the heterogeneity in TS would likely improve classification.

### Our results are consistent with atypical, not immature, functional connectivity in TS

While SVM was able to significantly distinguish children with and without TS, we did not find evidence that this discriminability was due to immature functional connectivity. Using SVR to predict age from RSFC data as in Dosenbach *et al*. (2010), we tested the predicted age for children with TS against a typical development maturation curve. The results demonstrated that the children with TS fell well within the curve, showing that the SVR successfully predicted age for children with TS similar to typically developing individuals. These results are apparently inconsistent with previous reports of immature connectivity in TS by us (Church *et al*., 2009a) and others (Worbe *et al*., 2012). This discrepancy may be due to the nature of the experiments, as we used a different approach to test for immaturity from previous studies. For example, in Church *et al*. (2009a), we examined two specific control systems, targeting a limited number of regions, whereas in the current study we examined a large number of regions covering much of the cerebral cortex. It is also possible that our implementation of improved methods for minimizing motion artifact in the present study underlies the discrepant results. If the previous reports of immaturity were due to motion artifact, when the effects of motion are better controlled the differences that remain between children with and without TS would reflect atypical functional connectivity (Church *et al*., 2009a).

### Limitations

Ideal confirmation of the present results will require independent replication. However, three different tests support their validity. First, the LOOCV method showed significant classification accuracy (~70%). This method has been shown to be reliable and provides a conservative estimate of the true expected accuracy (Pereira *et al*., 2009), and given our sample size, it is a useful method for obtaining an unbiased estimate of the classifier's generalizability. Second, we performed an experimental ‘control’ SVM to check the likelihood that the LOOCV approach artificially inflated accuracy. For this analysis, we divided the children with and without TS into two groups, each composed of half of the TS children and half of the control children, and then tested whether SVMs could classify each child as belonging to group 1 or group 2. This non‐diagnosis‐based classifier performed at chance, demonstrating that our methods did not find differences between groups in which there should not be any true biological differences. Third, permutation analysis of the class labels demonstrated that the classification accuracy we obtained was on the tail of the distribution, suggesting that it was not likely due to chance.

An additional limitation is that the 264 ROIs from Power *et al*. (2011) sample subcortical structures sparsely. Given previous evidence implicating striatal systems in TS, further study may benefit from a better sampling of subcortical regions in addition to full cortical coverage.

Another point of consideration is that children with TS in the present study were not excluded for current psychoactive medications or comorbid neuropsychiatric disorders. There is some evidence suggesting that medications, particularly stimulants, can affect functional connectivity results (Mueller, Costa, Keeser, Pogarell, Berman *et al*., 2014). Thus, it is possible that our classifier detected medication‐induced functional connectivity differences between the groups rather than connectivity differences due to diagnosis. However, an examination of the individual subject data revealed that 68% of the 22 unmedicated TS children were classified with greater than 80% individual accuracy (Table S1). Further, there were no significant differences between medicated and unmedicated TS children in individual accuracy or in the proportion of children classified correctly (with individual accuracy > 60%). Thus, it is unlikely that successful classification was driven by the medicated children. Similarly, we examined the individual subject data with respect to comorbidities, and 71% of the TS children without comorbid conditions were classified with greater than 80% individual accuracy. Given the large proportion of our TS sample with comorbid ADHD, we tested specifically for differences between TS children with and without ADHD, and again found no significant differences in individual accuracy or in the proportion of children classified correctly (with individual accuracy > 60%). Thus, examination of individual child characteristics suggests that correct classification was not driven by medication status or comorbid conditions. Of course, these conclusions are limited by the use of parent‐report for assessing neuropsychiatric conditions and medication status in the control group, as self/parent‐report is not always accurate or complete. Our finding that 12 control children were almost always misclassified could reflect inaccurate diagnostic history. Future studies that collect more complete clinical data on all participants (patients and controls) will improve confidence in diagnoses.

An additional limitation of our study is the sample size, as SVMs work best with large samples. Thus, it is possible that we could achieve higher classification accuracy with more participants; however, we are unaware of a proper power analysis for SVM to quantify the sample size needed. Furthermore, our analyses were conducted on a limited amount of RSFC data per participant (~5 minutes) in order to equate data quantity across all participants. Interestingly, 15–20 minutes of RSFC data per participant has been recommended for individual subject functional connectivity estimates and diagnostic classification (Anderson, Ferguson, Lopez‐Larson & Yurgelun‐Todd, 2011a). Further, compelling evidence has recently shown respectable reproducibility with 9 minutes of data, with significant improvement as the amount of data increases to 27 minutes (Laumann, Gordon, Adeyemo, Snyder, Joo *et al*., 2015).

Finally, while we found highly significant group classification accuracy, accuracy greater than 70% is desirable for direct clinical application. Of note, our binary classification approach did not account for the clinical heterogeneity in TS, and it is likely that the heterogeneity in our sample reduced our ability to obtain higher accuracy. Thus, we would expect that a study with much larger samples that can account for the clinical heterogeneity of TS would demonstrate improved accuracy. There are several other potential ways to improve classification accuracy that may benefit the practical applicability of these methods, including collecting more data, combining multiple imaging data types (e.g. RSFC and structural MRI measures), and including additional clinical and behavioral information. However, although imperfect for clinical purposes, the demonstration of significant diagnostic accuracy with RSFC data is valuable for (a) demonstrating feasibility of the approach, (b) exploring the features that best discriminate the groups for future hypothesis generation, and (c) future application of the methods for predicting clinical outcome or treatment response.

### Conclusions

The results of the present study demonstrate, as a first test case, that RSFC data contain information for discriminating children with and without TS. With further refinement of the methods, this technique holds promise for predicting prognosis and treatment outcome for individuals with TS. Given the heterogeneity of the disorder, and the uncertain developmental trajectory for individuals, a classification tool like that used here may be quite powerful in aiding individualized predictions. Interestingly, at least 10–25% of children have tics at some point during childhood (Cubo *et al*., 2011; Snider *et al*., 2002; Stefanoff, Wolanczyk, Gawrys, Swirszcz, Stefanoff *et al*., 2008). However, the prevalence of Tourette's Disorder and Chronic Tic Disorder is estimated at 1–3% (Cubo *et al*., 2011; Khalifa & von Knorring, 2003; Scahill *et al*., 2014). Therefore, for most children with tics, symptoms improve and they do not go on to develop a chronic disorder. Given this epidemiology, when a child first presents with tics clinicians will often tell parents that their child's tics are likely to remit. Since the DSM diagnosis of TS requires tics to have been present for at least one year, clinicians may recommend returning to seek treatment only if the tics continue for one year. However, many parents are anxious to know whether or not their child will develop a chronic disorder. Further, children with tics who will go on to develop TS may benefit from early intervention. Therefore, a tool that can predict whether an individual child is likely to develop TS would have high clinical utility. Predicting treatment response for an individual would also be clinically beneficial, as the best available treatments for tics meaningfully improve symptoms in only about half of those treated – but not necessarily the same half for each treatment. Here, we present a first step towards this goal of individualized predictions, demonstrating that brain imaging data, specifically RSFC, can be used for diagnostic classification. These results are promising: with further refinement of the methods to improve classification accuracies, application to future longitudinal datasets could test for predictions of symptom progression and responses to treatment. In addition, the methods used here could be applied to other neurodevelopmental disorders that would also benefit from individualized predictions.

## Supporting information


**Figure S1** Permutation of the class/group labels shows that the 74% diagnostic classification accuracy obtained for the 264‐region SVM with 400 features was not due to chance. Histogram bars indicate the number of permutations that resulted in each classification accuracy bin.

**Figure S2** RSFC connection and region weights. Functional connections driving the TS vs. control SVM classifier are displayed on a surface rendering of the brain. The thickness of the connections scale with their weights and the color of the connections indicate which group had higher correlations. The size of the ROIs also scale with their weights (1/2 sum of the weights of all the connections to and from that ROI).

**Figure S3** RSFC maturation curve for typical development (blue, *n* = 129). SVR predicts the age of children with TS (red) similarly to the typical developmental sample. Predicted age for children with TS represents the mean predicted age from 129 SVR models generated from the typical developmental sample. Error bars indicate the 95% confidence interval for each TS participant.

**Table S1.** Individual participant characteristics
