# Supplementary figures and images for: Multivariate pattern classification of pediatric Tourette syndrome using functional connectivity MRI
Source: Dev Sci. 2016 Feb 1;19(4):581–98. doi: 10.1111/desc.12407 (PMC4945470; doi:10.1111/desc.12407)

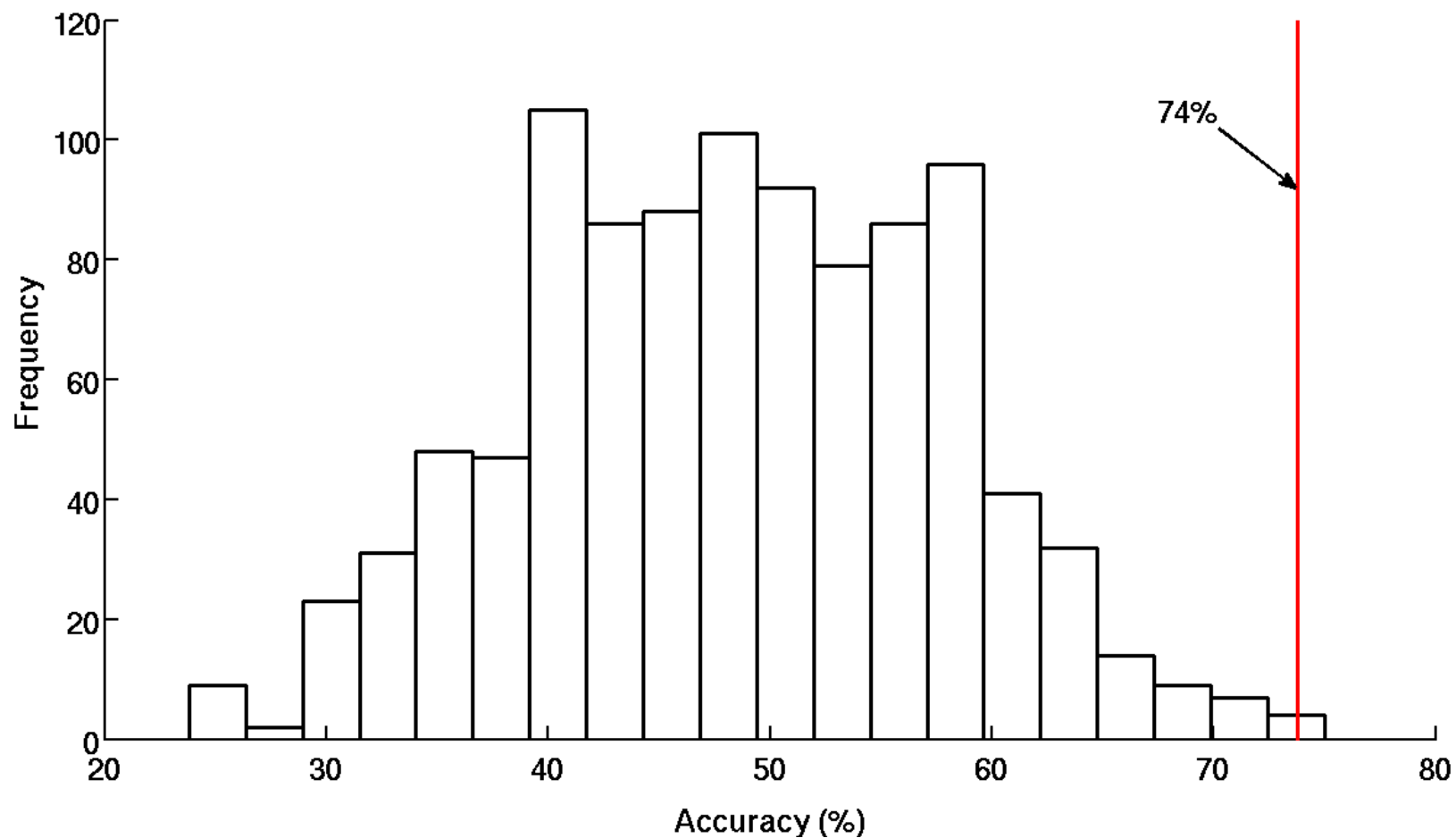

Supplement: Supplementary file 1 — Figure S1 Permutation of the class/group labels shows that the 74% diagnostic classification accuracy obtained for the 264‐region SVM with 400 features was not due to chance. Histogram bars indicate the number of permutations that resulted in each classification accuracy bin. [file DESC-19-581-s001.pdf]

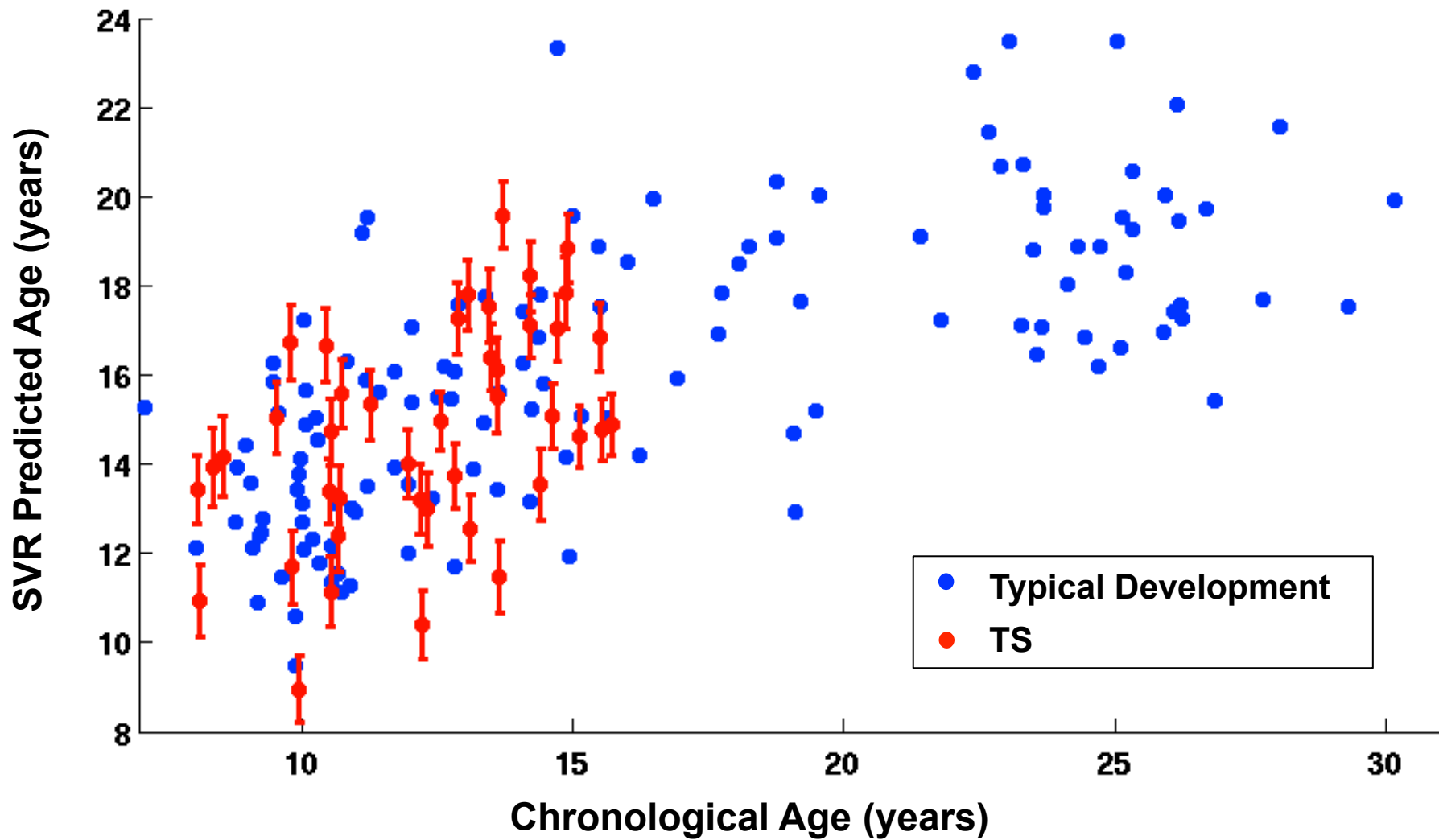

Supplement: Supplementary file 3 — Figure S3 RSFC maturation curve for typical development (blue, n = 129). SVR predicts the age of children with TS (red) similarly to the typical developmental sample. Predicted age for children with TS represents the mean predicted age from 129 SVR models generated from the typical developmental sample. Error bars indicate the 95% confidence interval for each TS participant. [file DESC-19-581-s003.pdf]
