# Supplementary material for: Multivariate pattern classification of pediatric Tourette syndrome using functional connectivity MRI
Source: Dev Sci. 2016 Feb 1;19(4):581–98. doi: 10.1111/desc.12407 (PMC4945470; doi:10.1111/desc.12407)
Supplement: Supplementary file 2 — Figure S2 RSFC connection and region weights. Functional connections driving the TS vs. control SVM classifier are displayed on a surface rendering of the brain. The thickness of the connections scale with their weights and the color of the connections indicate which group had higher correlations. The size of the ROIs also scale with their weights (1/2 sum of the weights of all the connections to and from that ROI). [file DESC-19-581-s002.pdf]

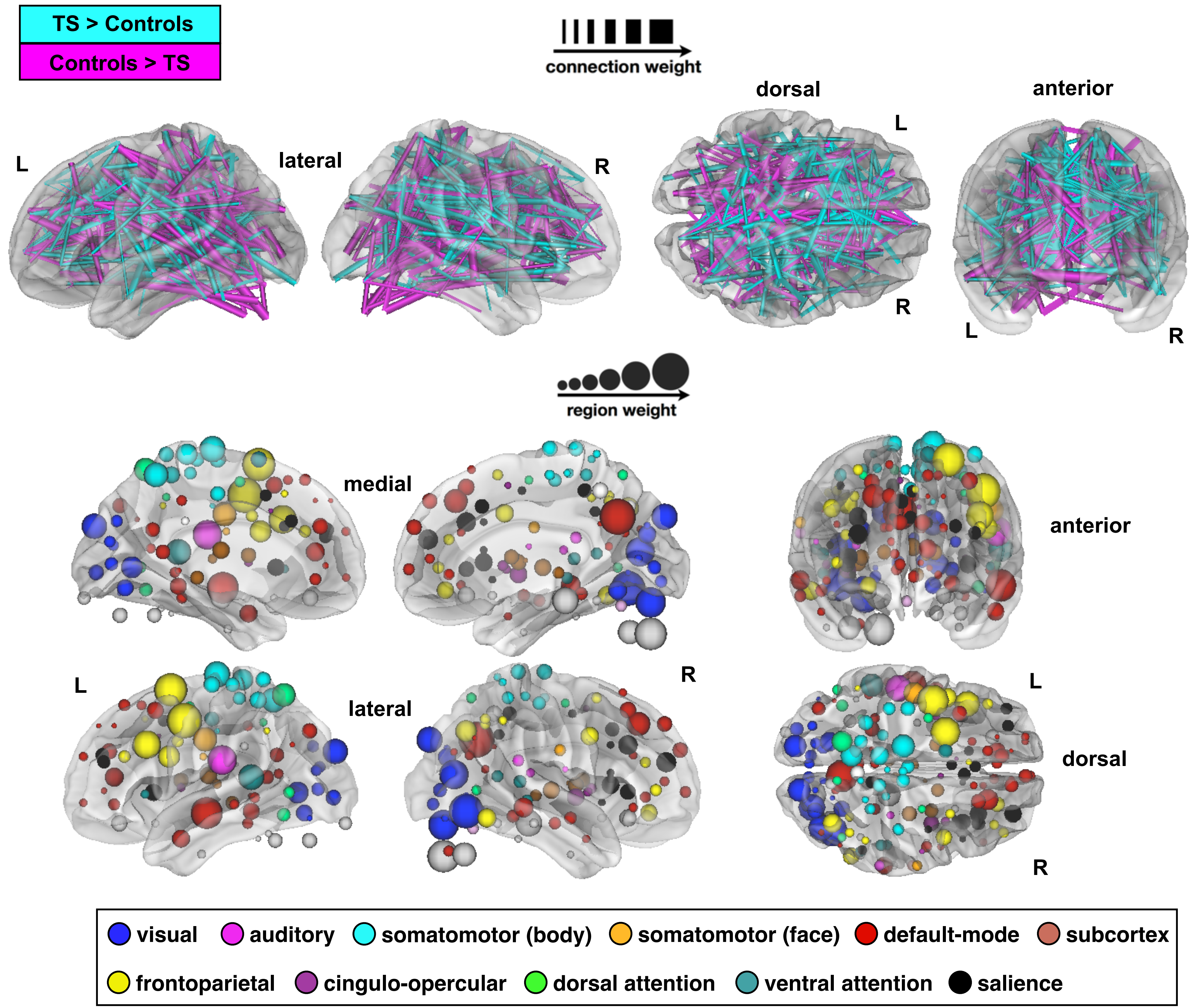
TS > Controls
Controls > TS
connection weight
lateral
dorsal
anterior
L
R
region weight
medial
lateral
anterior
dorsal
visual
auditory
somatomotor (body)
somatomotor (face)
default-mode
subcortex
frontoparietal
cingulo-opercular
dorsal attention
ventral attention
salience
