# Supplementary material for: Multivariate pattern classification of pediatric Tourette syndrome using functional connectivity MRI
Source: Dev Sci. 2016 Feb 1;19(4):581–98. doi: 10.1111/desc.12407 (PMC4945470; doi:10.1111/desc.12407)
Supplement: Supplementary file 4 — Table S1. Individual participant characteristics [file DESC-19-581-s004.docx]

Table S1. *Individual participant characteristics*

| Participant | Age | Sex | IQ | Handedness | Mean FD | YGTSS Total Tic Score | CY-BOCS Score | ADHD Rating | Comorbidities | Medications  (Y/N) | Individual  Accuracy (%) |
| --- | --- | --- | --- | --- | --- | --- | --- | --- | --- | --- | --- |
| TS01 | 13.1 | M | 101 | R | 0.1104 | 6 | 14 | 12 | ADHD | Y | 100.0 |
| TS02 | 10.7 | M | 112 | R | 0.1021 | 9 | 4 | 32 | ADHD | N | 80.7 |
| TS03 | 14.7 | F | 109 | R | 0.1054 | 17 | 0 | 5 | None | N | 100.0 |
| TS04 | 14.9 | F | 125 | L/A | 0.0857 | 13 | 0 | 1 | None | Y | 100.0 |
| TS05 | 13.1 | M | 135 | R | 0.0966 | 0 | 0 | 14 | None | Y | 100.0 |
| TS06 | 9.8 | M | 126 | R | 0.1117 | 15 | 0 | 12 | ADHD | Y | 100.0 |
| TS07 | 8.4 | F | 116 | R | 0.1093 | 16 | 0 | 10 | None | Y | 0.0 |
| TS08 | 10.5 | M | 87 | R | 0.1067 | 21 | 0 | 14 | ADHD | N | 100.0 |
| TS09 | 12.0 | M | 125 | R | 0.0914 | 8 | 6 | 17 | ADHD | Y | 80.7 |
| TS10 | 13.5 | M | 104 | R | 0.0968 | 26 | 0 | 15 | ADHD | Y | 100.0 |
| TS11 | 10.0 | M | 122 | R | 0.0982 | 33 | 0 | 25 | ADHD | Y | 89.2 |
| TS12 | 12.6 | M | 108 | R | 0.1042 | 15 | 6 | 1 | None | N | 100.0 |
| TS13 | 15.5 | M | 89 | R | 0.1158 | 16 | 1 | 12 | ADHD, tremor | Y | 100.0 |
| TS14 | 14.2 | M | 118 | R | 0.0983 | 8 | 0 | 8 | ADHD, dyscalculia | N | 98.8 |
| TS15 | 12.8 | M | 99 | R | 0.0993 | 10 | 0 | 6 | None | N | 2.4 |
| TS16 | 14.9 | M | 125 | R | 0.0705 | 11 | 0 | 0 | None | N | 90.4 |
| TS17 | 13.7 | M | 107 | R | 0.0810 | 14 | 0 | 4 | Past OCD, anxiety | N | 100.0 |
| TS18 | 14.6 | M | 98 | R | 0.1092 | 26 | 8 | 26 | ADHD | Y | 95.2 |
| TS19 | 13.7 | M | 94 | R | 0.1069 | 12 | 0 | 11 | None | N | 20.5 |
| TS20 | 13.6 | M | 105 | R | 0.0979 | 6 | 0 | 1 | None | N | 63.9 |
| TS21 | 12.3 | M | 110 | R | 0.0853 | 10 | 4 | 25 | ADHD | Y | 56.6 |
| TS22 | 10.6 | M | 117 | R | 0.0823 | 23 | 4 | 15 | None | N | 100.0 |
| TS23 | 13.6 | M | 99 | R | 0.0898 | 12 | 0 | 1 | None | Y | 100.0 |
| TS24 | 12.9 | M | 93 | L | 0.1077 | 8 | 0 | 13 | ADHD, developmental delay | Y | 4.8 |
| TS25 | 10.8 | M | 122 | R | 0.0948 | 26 | 8 | 6 | None | N | 88.0 |
| TS26 | 15.7 | M | 97 | R | 0.1046 | 21 | 0 | 5 | Subthreshold ADHD | Y | 100.0 |
| TS27 | 10.5 | M | 112 | R | 0.0963 | 18 | 2 | 11 | ADHD, OCD | Y | 83.1 |
| TS28 | 10.7 | M | 131 | R | 0.1199 | 17 | 0 | 8 | None | N | 100.0 |
| TS29 | 13.5 | M | 89 | R | 0.0973 | 32 | 20 | 34 | ADHD, OCD | N | 100.0 |
| TS30 | 15.1 | F | 124 | R | 0.0904 | 27 | 12 | 4 | Insomnia, depression, past ADHD | Y | 100.0 |
| TS31 | 14.2 | M | 117 | R | 0.0824 | 13 | 4 | 8 | ADHD, OCD | Y | 100.0 |
| TS32 | 11.3 | M | 118 | R | 0.1118 | 32 | 12 | 17 | ADHD, OCD | Y | 55.4 |
| TS33 | 8.6 | M | 108 | R | 0.1135 | 15 | 0 | 34 | ADHD | Y | 100.0 |
| TS34 | 12.2 | F | 117 | R | 0.1040 | 11 | 6 | 13 | ADHD | Y | 100.0 |
| TS35 | 9.8 | M | 126 | R | 0.1272 | 23 | 12 | 9 | OCD | N | 4.8 |
| TS36 | 8.1 | F | 133 | R | 0.1234 | 6 | 19 | 11 | ADHD, OCD | N | 100.0 |
| TS37 | 12.2 | M | 111 | R | 0.1031 | 14 | 15 | 4 | Subthreshold OCD, learning disability, sensory hypersensitivity | N | 100.00 |
| TS38 | 10.5 | M | 99 | R | 0.1041 | 17 | 0 | 0 | None | N | 100.0 |
| TS39 | 9.5 | M | 114 | R | 0.1189 | 22 | 16 | 2 | None | N | 98.8 |
| TS40 | 14.4 | F | 128 | R | 0.0974 | 10 | 0 | 27 | ADHD | N | 49.4 |
| TS41 | 15.6 | F | 105 | R | 0.0947 | 27 | 22 | 6 | OCD | N | 36.1 |
| TS42 | 8.1 | M | 104 | R | 0.1189 | 15 | 0 | 18 | None | N | 0.0 |
| CTL01 | 12.0 | M | 111 | R | 0.1024 |  |  |  |  |  | 16.9 |
| CTL02 | 15.0 | F | 104 | R | 0.1076 |  |  |  |  |  | 0.0 |
| CTL03 | 15.0 | M | 108 | R | 0.0734 |  |  |  |  |  | 0.0 |
| CTL04 | 12.4 | M | 108 | R | 0.0608 |  |  |  |  |  | 95.2 |
| CTL05 | 12.8 | M | 110 | R | 0.0695 |  |  |  |  |  | 0.0 |
| CTL06 | 15.0 | M | 129 | R | 0.0810 |  |  |  |  |  | 100.0 |
| CTL07 | 13.4 | M | 125 | R | 0.0950 |  |  |  |  |  | 7.2 |
| CTL08 | 11.0 | M | 117 | R | 0.1084 |  |  |  |  |  | 80.7 |
| CTL09 | 10.1 | M | 112 | R | 0.1151 |  |  |  |  |  | 100.0 |
| CTL10 | 12.0 | M | 122 | R | 0.0939 |  |  |  |  |  | 100.0 |
| CTL11 | 14.1 | M | 115 | R | 0.1121 |  |  |  |  |  | 26.5 |
| CTL12 | 13.4 | F | 105 | R | 0.0825 |  |  |  |  |  | 26.5 |
| CTL13 | 9.2 | M | 121 | R | 0.1036 |  |  |  |  |  | 1.2 |
| CTL14 | 10.7 | M | 128 | R | 0.1099 |  |  |  |  |  | 100.0 |
| CTL15 | 14.4 | F | 95 | R | 0.0975 |  |  |  |  |  | 100.0 |
| CTL16 | 14.5 | M | 114 | R | 0.1179 |  |  |  |  |  | 98.8 |
| CTL17 | 12.4 | M | 120 | R | 0.1051 |  |  |  |  |  | 98.8 |
| CTL18 | 11.3 | M | 126 | R | 0.1012 |  |  |  |  |  | 100.0 |
| CTL19 | 8.8 | M | 101 | R | 0.1062 |  |  |  |  |  | 94.0 |
| CTL20 | 11.2 | M | 121 | R | 0.1033 |  |  |  |  |  | 2.4 |
| CTL21 | 14.3 | F | 86 | R | 0.0818 |  |  |  |  |  | 22.9 |
| CTL22 | 15.0 | M | 123 | R | 0.1196 |  |  |  |  |  | 69.9 |
| CTL23 | 14.7 | M | 115 | R | 0.0655 |  |  |  |  |  | 95.2 |
| CTL24 | 13.7 | M | 107 | R | 0.1258 |  |  |  |  |  | 0.0 |
| CTL25 | 14.6 | F | 125 | R | 0.1019 |  |  |  |  |  | 100.0 |
| CTL26 | 13.2 | M | 107 | R | 0.0739 |  |  |  |  |  | 100.0 |
| CTL27 | 11.6 | M | 106 | R | 0.0961 |  |  |  |  |  | 100.0 |
| CTL28 | 15.3 | F | 101 | R | 0.0928 |  |  |  |  |  | 100.0 |
| CTL29 | 12.0 | M | 98 | R | 0.0999 |  |  |  |  |  | 0.0 |
| CTL30 | 10.3 | M | 108 | R | 0.0881 |  |  |  |  |  | 0.0 |
| CTL31 | 10.0 | M | 103 | R | 0.1181 |  |  |  |  |  | 55.4 |
| CTL32 | 8.7 | M | 111 | R | 0.1233 |  |  |  |  |  | 100.0 |
| CTL33 | 12.6 | M | 114 | R | 0.0797 |  |  |  |  |  | 0.0 |
| CTL34 | 9.9 | M | 122 | R | 0.0921 |  |  |  |  |  | 100.0 |
| CTL35 | 10.3 | M | 97 | R | 0.0980 |  |  |  |  |  | 100.0 |
| CTL36 | 10.2 | M | 98 | R | 0.0926 |  |  |  |  |  | 28.9 |
| CTL37 | 9.9 | M | 118 | R | 0.1135 |  |  |  |  |  | 100.0 |
| CTL38 | 9.3 | F | 96 | R | 0.1111 |  |  |  |  |  | 100.0 |
| CTL39 | 12.8 | M | 124 | R | 0.1065 |  |  |  |  |  | 98.8 |
| CTL40 | 8.7 | F | 109 | L | 0.1169 |  |  |  |  |  | 0.0 |
| CTL41 | 10.1 | M | 108 | L | 0.1103 |  |  |  |  |  | 1.2 |
| CTL42 | 12.1 | M | 136 | R | 0.0808 |  |  |  |  |  | 100.0 |

CTL = Control participant; FD = framewise displacement (mm) post volume censoring

Control group was not administered the YGTSS, CY-BOCS, or ADHD Rating Scale

All Control participants reported no neuropsychiatric conditions and no psychoactive medications

Individual accuracy is derived from the leave-two-out-cross-validation approach reported in Methods
