# Supplementary material for: Multivariate pattern classification of pediatric Tourette syndrome using functional connectivity MRI
Source: Dev Sci. 2016 Feb 1;19(4):581–98. doi: 10.1111/desc.12407 (PMC4945470; doi:10.1111/desc.12407)
Supplement: Supplementary file 5 [file DESC-19-581-s005.docx]

**RESPONSE TO REVIEWERS**

We thank the reviewers for their constructive comments and suggestions. The reviews were both very insightful and helpful. We considered all comments carefully in our revisions.

Regarding the editor’s suggestion that we particularly address the issue of mechanism, we realized the need to clarify the purpose of the study as well as the limitations of the method. The major aim of this study was to assess whether or not resting-state functional connectivity (RSFC) brain data contains sufficient discriminating information to predict whether a given child has TS or not. We tested for this discrimination using support vector machine (SVM) classification, which is well suited for making these types of predictions. While we can examine the SVM feature weights to identify the key features that, in aggregate, contribute to the classification, this ultimately is not a hypothesis driven method that can answer questions about mechanisms per se. This approach can provide clues about the brain regions/networks involved, which can guide future testable hypotheses, but claims about the underlying biological mechanism cannot be made here. Our findings demonstrate that there is information in the patterns of functional connectivity across many brain systems, thereby demonstrating the promise of using RSFC for individualized predictions. However, identification of specific biological mechanisms underlying TS will need a hypothesis driven approach. We hope that our changes to the manuscript now clarify this point. We now reword parts of the Introduction so as not to set up expectations about mechanism (pgs. 4-5) and clarify our study goals (pg. 8). We also now explain the limitation of this method for identifying mechanism and modify the text accordingly in the Discussion (pg. 24).

We also now emphasize our novel adaptation of SVM classification in this manuscript that estimates confidence of classification accuracy for each individual. We now call this estimate “individual accuracy” throughout the manuscript. We have added a statement to the Abstract and created a separate sub-heading in the Methods (pg. 15).

Please note the addition of a supplemental figure, which changed the numbering for all supplemental figures.

Specific changes to the manuscript in response to each point raised by the reviewers, and where they occur, are listed below.

**REVIEWER 1:**

*1. The authors discuss the significant clinical heterogeneity in Tourette syndrome with regard to presentation and outcomes. Indeed, one of the stated goals of the report is to use these data as “springboard” to clinical utility by predicting future differing outcomes. Yet the approach and discussion considers the phenotype as resulting from one mechanism. The authors may wish to discuss how the described heterogeneity in this population fits with their current model.*

We thank the reviewer for this suggestion. We now clarify that while TS is indeed heterogeneous, this paper presents a first step that does not account for the heterogeneity (added to the Introduction pg. 8). In fact, we believe that the clinical heterogeneity in TS may have reduced our ability to obtain higher accuracy than ~70%. However, our ability to classify with significant accuracy even without accounting for the heterogeneity demonstrates the power of the method for individual prediction (added to the Discussion pg. 29).

In addition, even though we ran binary classification (TS vs. control), we do not mean to suggest that TS is a single phenotype or results from a single mechanism. Rather, there are likely overlapping features that link these different individuals with TS together; the nature of those common features likely represent multiple underlying mechanisms. Here we examine neuroimaging features that drive a classification to separate TS from controls, but we do not mean for this examination to be a demonstration of a mechanism. As discussed above, these features can provide clues about the underlying common neurobiology, but only a hypothesis driven approach can directly test for the specific mechanisms involved in TS (or subgroups of TS) (added to the Discussion, pg. 26).

*2. The Fair et al paper cited here, prior to using classification in the ADHD population, did extensive testing on several different methods for removing artifact and movement, including the approach used in the current report. As such, it shouldn’t be “lumped” with these other reports, which did not account for this type of artifact. That should be qualified in this paragraph, which suggests it did not account for the same type of movement corrections that are applied here.*

The reviewer makes a good point about the Fair et al paper. The sentence referenced by the reviewer has been modified to make it clear that Fair et al. was an exception to the others (pg. 7).

*3. As functional connectivity has been shown to be sensitive to medication status (particularly stimulants), a discussion of how this might affect results should be described more clearly. Showing that classification accuracy can be equivalently shown when the analysis is conducted on participants without medications would help with this issue as well.*

We have expanded the paragraph on medications and comorbidities in the Limitations section of the Discussion to discuss more thoroughly the potential effects of medications. We are hesitant to conduct additional SVM analyses using only unmedicated TS participants, as the sample size would be reduced by nearly half. With 22 unmedicated TS children, we do not expect to have adequate power for classification (see response #6 to Reviewer 2 regarding power). However, we can take a first order approach to examine the influence of medication status by looking at individual subject classification accuracy (Table S1). Of the unmedicated TS children, 68% of them were classified with greater than 80% individual accuracy, suggesting that medications were not driving higher classification accuracies. We also took a statistical approach to address 2 alternative faces of this concern. Medications could “normalize” brain activity, in which case including medicated subjects may reduce our power to detect group differences, and we would expect classification accuracy to be lower in the medicated subjects. Alternatively, and more problematically, medication could induce changes in brain activity, enhancing group differences, and we would expect classification accuracy to be higher in the medicated subjects. The data in Table S1 reveal no significant differences between the medicated and unmedicated TS subjects in terms of mean classification accuracy (74.3 ± 37.1% meds, 83.3 ± 30.9% no meds, p=0.40, 2-tailed t test) and in terms of proportion of subjects classified correctly, i.e., with accuracy >60%, (16/22 meds, 16/20 no meds, p=.58, χ^2^ test). Of course these conclusions are limited by reasonable assumptions made about treatment status in the control group, which was based on parent report. This information and analyses have been added to the Results (pg. 19) and to the Discussion (pg. 28).

*4. Along the same lines, a large number of participants have ADHD and or other co-morbidities, which questions the specificity of the findings. As similar results have been shown in other clinical populations, could a similar analysis (i.e. LOOCV and/or LTOCV) be conducted on those participants that are TS only to show that the results do not change?*

Similar to the previous response, we examined the individual subject classification accuracy for those subjects who did not report other diagnoses (n=17). Of these children, 71% of them were classified with greater than 80% individual accuracy. This high proportion of children without comorbidities classified successfully suggests the overall classifier was not driven by comorbid conditions. Further, we ran similar t-test and χ^2^ test analyses to compare classification accuracy between TS subjects with and without ADHD. These analyses showed no significant differences between the TS subjects with and without ADHD in terms of mean classification accuracy (84.7 ± 25.1% with ADHD, 74.4 ± 40.5% without ADHD, p=0.37, 2-tailed t test) and in terms of proportion of subjects classified correctly, i.e., with accuracy >60%, (16/20 with ADHD, 13/17 without ADHD, p=.79, χ^2^ test). This information and analyses have been added to the Results (pg. 19-20) and to the Discussion (pg. 28).

*5. The methods read as if there were two slightly unique resting connectivity-preprocessing procedures conducted, is this correct?*

There were not two different RSFC preprocessing procedures. We have now added a sentence to the beginning of the “Preprocessing” section (pg. 11) and rephrased the first sentence of the “Functional connectivity preprocessing” section (pg. 11) to make it clear that all subjects were processed with the same approach.

*6. I really appreciated the permutation analyses run to identify the level of chance for this type of analysis. It is important when running LOOCV. However, I didn’t understand why the authors didn’t randomize labels of the entire set and re-run the analysis (with several permutations) to identify chance levels. As it reads now it looks as if the analysis was run on one(?) random 21 participant sample instead of the entire sample, which would have identical sample size and data characteristics as the original sample.*

We ran one random experimental control SVM using the entire sample of subjects. We pseudo-randomized the group labels to create 2 matched groups of 42 in which each group consisted of 21 TS and 21 control subjects. Thus the SVM was to classify Group 1 (21 TS + 21 controls) vs. Group 2 (21 TS + 21 controls). We now clarify our language in the Methods (pgs. 14) and Results (pg. 18). Thanks to the reviewer’s comment, we also now include a new randomized permutation analysis of the subject labels (Methods pg. 14-15, Results pg. 18, new Figure S1 – note that the numbering of the supplemental figures has changed with the addition of this new supplemental figure). This permutation showed that the classification accuracy obtained from the TS vs. control SVM was on the tail of the distribution.

*7. The authors are examining feature weights from the SVM models, but ones ability to extract weights is typically from linear SVM. Is that what was conducted here? It is not clear from the methods. If not (i.e. if a radial basis function was used), these weights are likely not the weights from the models used to generate the classification accuracies. I’m not an expert, but this is something the authors might want to consider.*

Linear SVM was conducted here. We have added a sentence to the Methods to explicitly state that we used linear SVM (pg. 13).

*8. “These results are inconsistent with previous reports of immature connectivity in TS (Church, Fair, et al., 2009; Worbe et al., 2012). This discrepancy is likely due to our implementation of improved methods for minimizing motion artifact in the present study.”*

*Actually, this is a different test than the prior report. The interpretation of immaturity in the prior report was based on a long-range versus short-range connectivity distinction - not SVM. To be sure that the result of "mature, but different" distinction between the papers is accurate, one would want to take the consensus feature weights from the model, split them up by bigger in TS versus bigger in controls, and show that that prior long-range/short-range distinction does not exist here. If it does not, the following statement, which states that the discrepancy is due to new motion correction procedures, would be true (most likely). If not, the difference in the outcomes would be due to the approach/methods used (less likely), or both. To be sure, however, one would have to do the test.*

We now recognize that we overstated our interpretation of the data. Consequently, we now soften the language in the Discussion (pg. 26-27).

In our hands, once processing methods to minimize motion artifact are implemented, we no longer find evidence for increasing long-range connectivity and decreasing short-range connectivity with age over typical development. Thus, we are not inclined to examine long-range vs. short-range connections in order to address the question of immaturity in this manuscript.

**REVIEWER 2:**

*This resting state analysis paper on TS vs normal children supports the conceptualization of TS as a broader disease involving more than just somatomotor regions.  This study suggests that TS is not a disease of global brain functional immaturity. Although this paper is a wonderful example of the power of multivariate analysis to detect group differences, the lack of a biological mechanism even in retrospect to explain the increased accuracy and to explain the consistently incorrect classifications for 20% of the subjects is disconcerting.  Overall, although the concept of using SVM to predict TS or predict evolution is intriguing and worth continued exploration, this paper does not prove that SVM can do this and the results are a little overstated. Vague words such as “high accuracy” and “most” subjects should be more precise and quantitative. In addition this study did not evaluate if SVM can predict who will develop TS or go on to have severe symptoms. In fact, in this study it the SVM cannot yet differentiate those with severe vs mild tics.*

The reviewer’s summary includes excellent points that we now address. We now clarify the purpose of the study and acknowledge that SVM methods are not well suited to answer questions about biological mechanisms (as also pointed out by the editor and Reviewer 1). We soften our language throughout the manuscript in order to not overstate our claims and provide precise numbers in place of vague statements. We also emphasize that this study is a first step toward individualized predictions in TS with several limitations. Regarding the inability to predict symptom severity in this study, there are a number of possible explanations that we discuss in response #12 below.

*1. The imaging acquisition is confusing in parts – “1 image for all controls and for 29 of the TS participants” should read “one sequence acquisition for each control subject and for each of 29 TS participants” and “2 images for 13 of the TS participants” should read “2 sequence acquisitions for each of the 13 remaining TS participants”.*

We have changed this sentence according to the reviewer’s suggestion (pg. 10).

*2. The total time of the resting state sequence should be stated.*

We have now included the length and number of resting state sequences: “For most participants (40 TS, 34 controls), 2-4 resting state sequences lasting 5-5.5 min each were acquired. The remaining 2 TS participants completed 3 sequences lasting 6.8 minutes each, and of the 7 remaining control participants, 4 of them completed 4 sequences lasting 3.2 minutes each and 3 of them completed 1 sequence lasting 30 minutes.” (pg. 10)

*3. Given that these are children, how did you ensure that they looked at the crosshair throughout the duration of the resting state scan?*

Resting state scans were conducted during relaxed fixation; children were not instructed to stare at the crosshair for the duration of the scan, but to “keep an eye on the plus sign” (now added to the Methods pg. 10). We did not monitor eye movements during data collection for the present study, so it is possible that there were systematic group differences in eye movement. However, there is no reasonable reason to assume that these groups, which are matched on age, sex, IQ, and in-scanner movement, would differ in the amount of eye movement. We, and others, currently collect data to monitor subjects’ behavior (including eye position) during resting state scans; however, these practices were not in effect when the data in the present study were acquired.

*4. Power et al. proposed a combined metric based on FD and DVARS. Some authors argue that DVARS is more accurate than FD, particularly in cases of extreme head motion. However, here it appears that motion assessment only takes into account FD.*

In a more recent paper from our lab (Power et al., 2014 *Neuroimage*), we show that FD alone is a perfectly sufficient metric to use when censoring volumes to reduce motion artifact. Thus, the methods in this manuscript follow those of Power et al. 2014.

*5. The atlas seems to be constructed from two populations with extremely different ages (7-9 yo, and 21-30 yo). This is confusing as a poor target atlas could introduce large errors. They include several references, but for completeness, it would be helpful to briefly explain the details in this paper.*

The atlas used in the present study has been used in many developmental fMRI studies (e.g., Fair et al. 2007, Fair et al. 2008, Church et al. 2008, Church et al. 2009), continues to be the preferred atlas for developmental studies in our lab, and has been used consistently in other labs, e.g., the Fair lab (personal communication). Its construction was motivated by the use of a combined young adult/older adult atlas for studies of aging in the Buckner lab (e.g., Logan et al. 2002, Lustig et al. 2003). Informal analysis in our lab demonstrates better fits across children and adults using the child/adult combined atlas. We have expanded our description of the construction of the atlas in the Methods (pg. 11). Most importantly, it is unlikely that use of this atlas would introduce systematic group bias in our study, as our subject groups were the same age.

*6. What were the sizes of the ROIs for the 264 regions? With spatial resolution of 4mm and filtering to 6mm FWHM it would be helpful to know.*

We thank the reviewer for pointing out this information that was missing from the methods. We used 10mm spheres, now added to the methods (pg. 12).

*7. Could you use SVM to determine which TS individuals had ADHD and or OCD features or those on meds vs not?*

Please see our responses #3 and #4 to Reviewer 1 on similar points. Unfortunately, our sample size is too small for definitive judgments about whether SVM can classify comorbidities or medication status, but individuals without these aspects were still well classified as TS vs. control.

*8. Selection of TS with at least 125 usable frames, resulted in only 47% of the TS population having usable data. Does filtering lead to a bias towards the less moving population and therefore decrease your ability to detect differences with worsening symptoms?*

We re-examined the total sample of TS subjects who enrolled in the study, and found that 6 of the 89 did not participate in the scan. Thus, 83 children with TS participated in all study procedures (changed in the Methods, pg. 9). We then examined the tic severity ratings for those children who were excluded due to motion. We found no difference in YGTSS Total Tic scores (i.e., motor + vocal scores) for the children who were included (mean = 16.5, SD = 8.6, range 0-39) vs. those who were excluded due to motion (mean = 17.2, SD = 8, range 0-34), *p* = 0.7. Thus, it does not appear that filtering biased the population to those children with less severe symptoms. We have added this comparison to the Results (pg. 17).

*9. What is the definition of “high accuracy”? Less than 1/3 of typical or TS subjects were classified with >70% accuracy and 29% of normal and 12% of TS were consistently misclassified with no clear reason why.*

We now include precise numbers and percentages (pg. 19). The individual subject classification shows that 64% of all subjects were classified with greater than 80% individual accuracy, and 45% of all subjects were classified with 100% individual accuracy.

Indeed, 29% of controls and 12% of TS subjects were consistently misclassified. However, many statistical tests have a false negative rate and diagnostic errors exist for any lab test. In the Limitations section of the Discussion, we offer speculation as to how to improve accuracy levels. In addition, we are not surprised that more controls were misclassified than TS subjects (12 controls, 5 TS). In recent data collection efforts in our lab, we have found that a number of children recruited as typically developing controls who pass our phone screen are later deemed ineligible because it is discovered that they have tics. This discovery is only made after lengthy clinical interviews with both the guardian and child. Thus, it is possible that a number of typically developing controls who did not undergo in depth clinical screening actually had tics that were unrecognized by the parent. Indeed, classroom observation studies find that at least 20% of children have tics at some point during childhood, yet only 1-3% of children are diagnosed with a chronic tic disorder. Thus, there are many cases of undiagnosed and likely unrecognized tics, current or past, which can contaminate control groups. We have added our own observation and the epidemiological numbers to the Discussion (pg. 23).

*10. SVM methods are data hungry. What is the minimum sample size needed to provide accurate classifications. Is there also a minimum number of BOLD frames needed for the same purposes.*

The question of sample size is a good one, and we are unaware of a definitive answer. We ran a degradation of our subject numbers to obtain an estimate of the sample size needed for accurate classification with our data. We degraded the number of subjects in each group by steps of 5 and calculated accuracy for each step, i.e., 42 vs. 42 (total sample), 37 vs. 37, 32 vs. 32, etc. We found that accuracy for classifying TS vs. controls remained ~70% until our sample was reduced to 22 vs. 22. With this sample size and smaller, accuracy dropped to 50-60%. Thus, it appears that we need at least 27 subjects in each group to obtain the expected accuracy. However, these calculations are far from a proper power analysis, and therefore, should not be included in the manuscript. With SVM analyses, we are unaware of a proper power analysis approach. Thus, the issue of sample size remains an open question. Since SVM methods are indeed data hungry, we might expect higher accuracy with much larger samples, but we cannot quantify power at this point.

Regarding a minimum number of frames needed for accurate classification, this question remains open as well. In the present study, we truncated each subject to 125 usable frames for the purpose of equating the groups, but this choice was not because we think 125 frames is all that is needed. There is compelling evidence supporting the need for more RSFC data, as at least ~10 minutes is needed to study individual region-to-region functional connectivity, while more than 30 minutes may be needed for more complex analyses with finer spatial specificity (Laumann et al. 2015, *Neuron*). Thus, we do believe that SVM methods would do even better with more data per subject, and the current manuscript represents an early effort to use this classification approach in the TS population.

We now discuss both issues of sample size and amount of RSFC data in the Limitations section of the Discussion (pg. 29).

*11. In the 12 subjects that were misclassified and given that the classification accuracy increases when the analysis is restricted to a subset of ROIs closely involved in TS, was a restricted number of ROIs attempted on these particular subjects?*

Using the full set of 264 ROIs, classification accuracy peaked at 74%. When using a restricted set of ROIs, the peak classification accuracy achieved was 73% (when including somatomotor and control systems). Thus, our targeted SVMs did not demonstrate improved accuracy compared to the full 264-ROI SVM.

*12. Was inability to predict tic severity related to the inclusion of only those with less severe motion?*

Please see response #8 above. The sample of TS subjects that were included did not have less severe symptoms than those subjects excluded due to motion in the scanner. The inability to predict tic severity may be due to several factors: YGTSS Total Tic score may not be an optimal measure of tic severity or it may not capture adequately tic severity during the moment of the scan (as it assesses tics during the “past week”), RSFC may not carry information about current tic severity, and with only 42 TS children we may not have adequate power to predict severity (pg. 20).

*13. The lack of clear differences in features separating normal and TS subjects (Figure S1) is challenging. Do you think spatial resolution of the resting state data may be an issue?*

To clarify, Figure S1 (now Figure S2) shows that there were many features separating TS and control subjects and that these features span many brain systems. The features demonstrate both stronger connectivity in TS than controls and stronger connectivity in controls than TS subjects. Thus, it is not that there is a lack of clear differences in the features. Rather, we cannot visually see a clear pattern in those differences. Given the complexity of TS, it is not necessarily surprising to us that the features are complex as well (pg. 24).

It is unlikely that resolution itself makes it difficult to assess features in resting state data. Many reports (Cohen et al. 2008 *Neuroimage*, Craddock et al. 2012 *Human Brain Mapping*, Wig et al. 2013 *Cerebral Cortex*, Gordon et al. 2014 *Cerebral Cortex*) show that resting state relationships are quite homogeneous over collections of several voxels. These observations have been used to suggest putative functional areas.  It is most likely the relationships between these areas that allow the SVMs to work.

*14. It is an overstatement to say that patterns of functional connectivity can classify children with and without TS when 40% of normal individuals and 19% of TS individuals are classified with accuracy < 50%. The technique shows promise but there is a ways to go. Although the largest cluster was accurately classified with 90-100% accuracy this was less than 50% of the total subjects.*

The reviewer is correct that our results represent a first step that shows promise, but is not a perfect demonstration of individualized prediction. Thus, we soften our language and include precise numbers throughout the Discussion. Our contention is that there is sufficient information in RSFC data to classify individuals with accuracy that is significantly higher than chance. In the Limitations section, we discuss that accuracy higher than 70% is desirable for clinical applicability, and we discuss ways in which accuracy might be improved (pg. 29). For example, we expect that with a deeper understanding of the heterogeneity in TS, RSFC data in aggregation with other data types (e.g., structural MRI) would yield improved accuracy.

*15. Although it is exciting that SVM can detect significant differences when traditional univariate approaches cannot, the lack of a clear biological meaning to the differences remains a challenge.*

This point by the reviewer again indicated our need to clarify the purpose of the study and our methods (as discussed above). Again, in brief, the main purpose of this study was to test how accurately RSFC could predict diagnosis on an individual subject level, and not to identify putative mechanisms. SVM methods are well suited for making these types of predictions, but are not ideal for identifying underlying mechanisms. Indeed, we can extract feature weights to examine those features that, in aggregate, contribute to the classification. While this examination can provide clues about the imaging features involved, it will not lead to an explanation of mechanism. In order to pin down a mechanism, one would need a hypothesis driven approach. The text has now been modified to better convey our study goals and the limitations of SVM for identifying mechanism (pg. 4-5, 8, 24).

*16. In particular the lack of any clear reason for misclassification in the 17/84 (20%) subjects is disconcerting.*

Please see above response #9 on the issue of misclassification.

*17. Given that most of the accuracy was obtained using somatomotor and higher level control systems could the global differences detected with SVM be driven by subtle systematic differences such as subtle motion differences?*

We think it is highly unlikely that residual motion artifact is driving systematic differences detected by the SVM. We implemented the most conservative methods to date to minimize motion artifact at the sub-millimeter level. Furthermore, the groups do not differ significantly in head movement even *after* motion censoring procedures (which is notable since motion censoring greatly reduces the variance). With our strategies for minimizing motion artifact, we do not believe motion is driving our results.

*18. It is unclear how much of the results were driven by OCD or ADHD comorbidity. Could isolated OCD or ADHD be differentiated from TS with these comorbidities? Could TS with ADHD be differentiated from TS with ADHD?*

Please see above responses #3 and #4 to Reviewer 1.

*19. The maturation index here appeared to be a whole brain approach with 264 regions and 1000 features whereas Church, Fair etc 2009, explored specific networks. A dominant normal maturation with only a subset of connections immature may not be detected by this SVM approach. Additional evidence would be necessary to support the statement that these prior results were due to motion alone as although larger motion was tolerated, the groups were matched. Was a subset of the same data sets analyzed here? With 6mm voxels would motion of 0.2 vs 1.7mm have a dramatic effect?*

Please see above response #8 to Reviewer 1. We overstated our interpretation of the results here, as we implemented a different approach to examine immaturity. We have made changes to the current manuscript accordingly. The data presented in the current study are separate from those in Church et al., 2009. Regarding motion, we have shown that sub-millimeter movements systematically affect functional connectivity results (Power et al., 2012; Power et al., 2014).

*20. A deeper discussion on the limitations and need for future prospective testing etc would be helpful. In particular a discussion on what features the SVM keys on to differentiate and if these can be biologically interpreted would be helpful to put this paper in context with biologically inspired treatments.*

Please see above response #15 and to the editor. We now re-word and include additional text to clarify the goal of our approach (prediction) and the limitations of examining SVM features for understanding biological mechanisms.

*21. Figure S1. Bottom two rows – It is unclear how TS and controls are compared. Which is which?*

Note that Figure S1 is now Figure S2. The size of the ROI nodes in the bottom of Figure S2 represent the ½ sum of the weights of all the connections to and from that ROI, including both those connections that are greater in TS and those that are greater in controls. Thus, the region weight does not distinguish the direction of the connection; information about the directionality is shown in the top row. We add clarification to the figure caption for Figure S2.
